# Symptom Provocation and Clinical Response to Transcranial Magnetic Stimulation: A Systematic Review and Meta-Analysis

**DOI:** 10.1001/jamapsychiatry.2025.0792

**Published:** 2025-06-04

**Authors:** Daniel Bello, Megan Jones, Ishaan Gadiyar, Laura Artim, Sophia H. Blyth, Roscoe O. Brady, Simon Vandekar, Heather Burrell Ward

**Affiliations:** 1 Department of Psychiatry and Behavioral Sciences, Vanderbilt University Medical Center, Nashville, Tennessee; 2 Department of Biostatistics, Vanderbilt University Medical Center, Nashville, Tennessee; 3 Department of Psychiatry, Harvard Medical School and Beth Israel Deaconess Medical Center, Boston, Massachusetts; 4 McLean Hospital, Belmont, Massachusetts

## Abstract

**Importance:**

Transcranial magnetic stimulation (TMS), a form of noninvasive brain stimulation used to treat major depressive disorder, obsessive-compulsive disorder (OCD), and nicotine dependence, has well-established state-dependent effects on brain circuitry. However, a major question for TMS remains: does brain state affect clinical response?

**Objective:**

To quantify the association between symptom provocation and clinical response to TMS for OCD and nicotine dependence, the only Food and Drug Administration–cleared TMS indications with symptom provocation.

**Data Sources:**

PubMed, CINAHL, Embase, PsycInfo until August 30, 2024.

**Study Selection:**

Randomized clinical trials of TMS for OCD or nicotine dependence with a clinical outcome. Of 600 studies identified, 71 met inclusion criteria.

**Data Extraction and Synthesis:**

Data extraction was completed independently by 2 extractors and cross-checked by a third. Standardized mean difference (SMD) and SE were estimated via Hedges *g* and synthesized data in a 3-level random-effects meta-analysis. Study data were analyzed from August 2023 to March 2025.

**Main Outcomes and Measures:**

Primary outcomes were clinical response measures.

**Results:**

A total of 71 studies met inclusion criteria and included 3246 participants (mean [SD] age; 37.8 [8.0] years; mean [SD] percentage female, 44.1% [17.2%]). Included in the meta-analysis were 63 studies with 2998 participants. For OCD studies, active TMS was associated with better clinical response than sham both with (SMD = −0.51; 95% CI, −0.96 to −0.07; *P* = 0.04) and without (SMD = −0.29; 95% CI, −0.40 to −0.17; *P* < .001) symptom provocation. For nicotine use, active TMS was associated with better clinical response than sham when used with (SMD = −0.56; 95% CI, −1.12 to 0; *P* = .05) but not without (SMD = −0.35; 95% CI, −0.74 to 0.04; *P* = .08) symptom provocation. For OCD studies, the estimated expected added effect of provocation was SMD of −0.22 (95% CI, −0.65 to 0.20; *P* = .22). In nicotine studies, the estimated expected added effect of provocation was SMD of −0.21 (95% CI, −1.00 to 0.58; *P* = .57).

**Conclusions and Relevance:**

Results of this systematic review and meta-analysis suggest that symptom provocation may enhance clinical response to TMS for OCD and nicotine dependence. Studies comparing TMS with and without provocation are critical to establish the causal effect of provocation.

## Key Points

**Question:**

What is the association between symptom provocation and efficacy of transcranial magnetic stimulation (TMS) for nicotine dependence and obsessive-compulsive disorder (OCD)?

**Findings:**

In this systematic review and meta-analysis of 71 studies (63 studies included in the meta-analysis), active TMS with symptom provocation had significantly better clinical response than sham for OCD and nicotine dependence; active TMS without provocation had significantly better response than sham for OCD but was nonsignificant for nicotine dependence. The stronger clinical effect with provocation was not significant.

**Meaning:**

Results of this study reveal that, although symptom provocation was not significantly better for OCD or nicotine dependence, the stronger effect sizes with symptom provocation suggest that further research is merited to accurately estimate effect strength.

## Teaser

This systematic review and meta-analysis investigates the association between symptom provocation and efficacy of transcranial magnetic stimulation (TMS) for nicotine dependence and obsessive-compulsive disorder.

## Introduction

Repetitive transcranial magnetic stimulation (TMS) is a form of noninvasive brain stimulation with US Food and Drug Administration (FDA) clearance for treatment of major depressive disorder, obsessive-compulsive disorder (OCD), and nicotine dependence. Although TMS is effective for many psychiatric and substance use disorders,^1,2,3^ questions remain about its underlying mechanism and ways to optimize clinical response.

It is well established that TMS has state-dependent effects on brain circuitry.^4,5,6,7^ State dependence signifies that the effect of brain stimulation is the result of the interaction between external stimulation and internal brain state.^8^ The state-dependent effects of TMS are readily observable during motor threshold determination, defined as the minimum stimulation level that will elicit a defined motor response (ie, usually a 50-mV motor-evoked potential or observable twitch in a defined muscle) in 50% of attempts. It is well established that active motor threshold (ie, the motor threshold when a muscle is partially contracted) is lower than the resting motor threshold.^9,10^ State-dependent effects of TMS have been demonstrated in the motor,^9^ visual,^11^ and memory systems^12^ where prestimulation neural activation increases susceptibility to TMS effects. This has also been shown for emotional states, where reactivation of a fear-related memory before TMS successfully eliminated physiological fear responses 24 hours after active TMS but not after sham TMS or when TMS was administered without fear reactivation.^13^ It has, therefore, been proposed that provoking symptoms may shift the brain into a state that is more susceptible to the effects of TMS, which may improve response.^8^ However, a major question for TMS treatment remains: does brain state affect clinical response?

**Figure 1.**
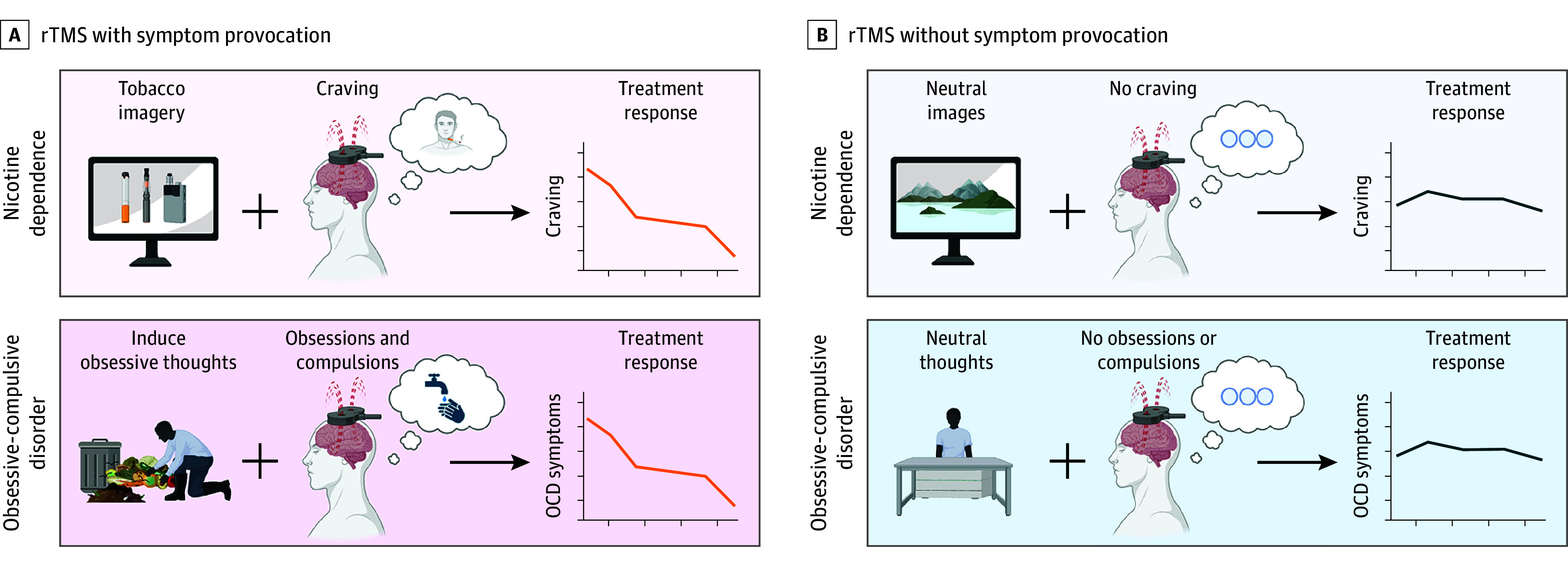
Model of Hypothesized Effects of Symptom Provocation on Clinical Response to Transcranial Magnetic Stimulation (TMS) Repetitive TMS (rTMS) has known state-dependent effects. The current US Food and Drug Administration–cleared TMS protocols for nicotine dependence and obsessive-compulsive disorder (OCD) both include provocation of a symptomatic state (craving, obsessive-compulsive thoughts, respectively) in an effort to improve TMS response. However, these experiments have been performed in small numbers, and the overall effect of symptom provocation has not been quantified. Figure created with BioRender.com.

The FDA-cleared TMS protocols for OCD and nicotine dependence provoke a symptomatic state before TMS in hopes of shifting the brain into a state more sensitive to TMS effects (Figure 1). For OCD, this involves provoking obsessive-compulsive symptoms through auditory or visual stimuli based on a personalized hierarchy for 5 minutes before TMS until their level of distress is 4 to 7 out of 10 on visual analog scale.^14,15^ Using this protocol, active TMS significantly reduced OCD symptoms with a response rate of 45.2% compared with 17.8% using sham.

For smoking cessation, the FDA-cleared protocol involves a 5-minute provocation procedure where patients imagine their greatest craving trigger, listen to an audio recording instructing them to handle a cigarette and lighter, then view smoking images.^16^ In the study that led to FDA clearance of TMS for smoking cessation, the active TMS group had a higher continuous quit rate (28.0% vs 11.7%) and greater reductions in cigarette consumption and craving compared with sham.

Evidence from other studies of TMS for smoking cessation,^17^ depression,^18^ posttraumatic stress disorder,^19^ and OCD^15,20,21^ suggests that provocation before or during TMS treatment may improve TMS response. However, this preliminary evidence has been accumulated from small studies, and this question has never, to our knowledge, been analyzed in sufficient numbers. Importantly, almost none of these studies, including the trials that led to FDA clearance, have done a direct comparison of TMS protocols with provocation and TMS protocols without provocation.

Therefore, it remains unknown if TMS protocols with symptom provocation improve clinical response more than protocols without provocation. Because the only FDA-approved TMS protocols that include symptom provocation are for OCD and nicotine dependence, we performed a systematic review and meta-analysis to estimate how much provoking a symptomatic state before TMS improves treatment response for these indications. Specifically, we aimed to aggregate effects across the field to estimate the effectiveness of active TMS compared with sham for OCD and nicotine dependence as well as the benefit of symptom provocation.

## Methods

### Protocol and Registration

This systematic review is reported in accordance with the Preferred Reporting Items for Systematic Reviews and Meta-analyses (PRISMA) reporting guidelines^22^ and is registered in the PROSPERO database (CRD42023390894).

### Eligibility Criteria

We restricted our search to articles that tested TMS for adults (at least 18 years of age) with OCD or nicotine dependence because these are the only currently FDA-cleared TMS protocols that include symptom provocation. Articles were included if they were (1) randomized clinical trials (RCTs) and (2) included a clinical outcome measure. The search strategy included descriptors for TMS, OCD, and nicotine dependence (eTable 1 in Supplement 1). For this systematic review, we defined a control group if it compared the following: (1) active TMS with sham TMS or an active control TMS target or (2) use of provocation with no provocation. Therefore, open-label trials of TMS without a control group were excluded.

### Outcomes

All clinical outcomes related to OCD symptoms and nicotine dependence were included (eg, Yale-Brown Obsessive-Compulsive Scale [YBOCS], craving, biological measures of nicotine use, Fagerstrom test for nicotine dependence [FTND], or cigarettes smoked per day).

### Search Strategy

We searched the following online databases: PubMed, CINAHL, PsycInfo, EBSCOhost (1872-present), and Embase on August 30, 2024. We included all studies that used TMS to treat OCD or nicotine dependence published in English. eTable 1 in Supplement 1 details the search strategy.

### Study Selection

Titles and abstracts were independently evaluated. Full-text articles were included if they met inclusion and exclusion criteria. All screens were performed by 2 separate reviewers (D.B., I.G.), with discrepancies resolved by a third (H.B.W.).

### Data Extraction

Three reviewers (D.B., I.G., L.A.) independently conducted the data extraction, and disagreements were resolved by a fourth reviewer (H.B.W.). General study characteristics were collected, including design, population, TMS parameters, and outcome measures. For the primary outcomes, pretreatment and posttreatment measures of clinical response (eg, YBOCS, nicotine craving) were collected for the active and sham groups.

### Qualitative Summary

Extracted data were analyzed in 2 steps: (1) qualitative summary and (2) meta-analysis. First, we qualitatively summarized the results of each article and performed a thematic analysis of patient characteristics, symptom provocation protocol, and main results. We extracted participant race when provided, but a majority of the studies did not provide details on race or ethnicity of participants. Therefore, only percentage of Black race is reported. Methodologic quality and risk of bias were assessed using the Cochrane Risk of bias tool.^23^ Risk of bias was determined as follows: (1) high risk when more than 1 indicator of bias was present across all scales and (2) low risk when 1 or no indicator of bias was present. Risk of bias was classified by 3 independent reviewers (D.B., I.G., L.A.), and differences were resolved by a fourth (H.B.W.).

### Strategy for Data Synthesis

For this analysis, the effect size quantifies the difference in an outcome measure comparing active with sham TMS treatment using Hedges *g* estimator of standardized mean difference (SMD).^24^ Thus, analyses were restricted to studies with a sham control condition. Outcome measures included YBOCS, Hamilton Anxiety Rating Scale, Beck Anxiety Inventory, FTND, cigarettes smoked per day, nicotine craving, cotinine:creatinine ratio, expired carbon monoxide, cue reactivity, time to relapse, and self-efficacy. The sign of the outcome variables was multiplied by −1 or 1, so that negative effect sizes indicate that active TMS treatment is associated with a greater reduction in symptoms than sham across all outcome variables. The method for estimating SMD for the difference between active and sham TMS interventions was selected based on the reported statistical values (eMethods in Supplement 1).

### Statistical Analysis

We performed a multilevel random-effects meta-analysis, which allowed us to include multiple estimates per study (eg, for different clinical outcomes) by accounting for within-study correlation using a random effect for study and to use study-specific random effects to account for additional variability between individual effect sizes due to heterogeneity in the effect across outcomes.^25^ Four meta-analytic models were estimated using the metafor R package (R Project for Statistical Computing). Model 1 was an intercept-only model quantifying the overall estimated effect size aggregated across the studies. Model 2 added a fixed effect for symptom provocation. Model 3 added a fixed effect comparing OCD vs nicotine studies with model 1. Model 4 included provocation, diagnostic group (ie, OCD, nicotine dependence), and their interaction. We used model 4 to estimate effect sizes and CIs for each relevant study group (eg, OCD studies with provocation) and to test for a significant provocation effect in either OCD or nicotine studies. As an exploratory analysis, we investigated the difference between excitatory and inhibitory TMS by adding a protocol covariate to model 4. Excitatory sequences were defined as any TMS greater than 1 Hz or intermittent theta burst stimulation, and inhibitory sequences were defined as any TMS of 1 Hz or less or continuous theta burst stimulation. For all models, we used the CR2 robust variance adjustment to account for variance misspecification within studies. We assessed within- and between-study heterogeneity using the *I*^2^ statistic and visually assessed evidence for publication bias using a funnel plot. We performed a sensitivity analysis that excludes the effect size estimates indicated as potential outliers by the funnel plot. Study data were analyzed from August 2023 to March 2025.

## Results

### Study Characteristics

**Figure 2.**
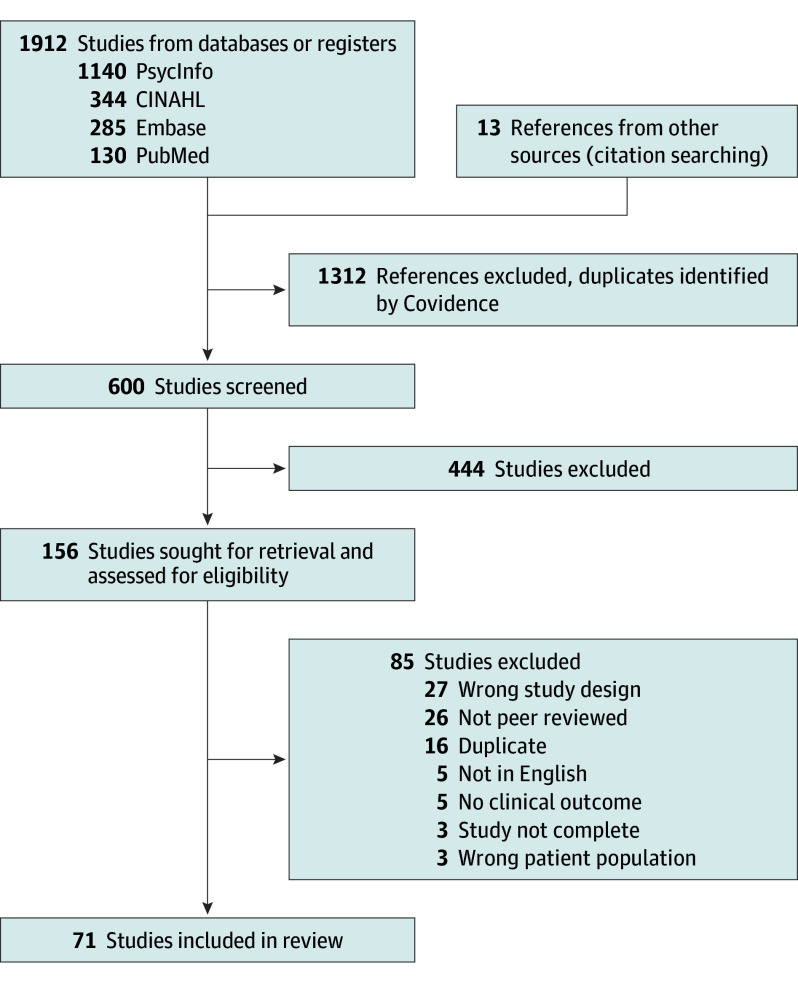
Preferred Reporting Items for Systematic Reviews and Meta-Analyses (PRISMA) Flow Diagram Shown here is the process of identification, review, and selection of articles included in the systematic review.

The initial search yielded 600 studies. After screening titles and abstracts, 156 full-text studies were assessed for eligibility. Seventy-one studies met inclusion criteria (n = 44 OCD^15,20,26,27,28,29,30,31,32,33,34,35,36,37,38,39,40,41,42,43,44,45,46,47,48,49,50,51,52,53,54,55,56,57,58,59,60,61,62,63,64,65,66,67^; n = 27 nicotine dependence^16,17,68,69,70,71,72,73,74,75,76,77,78,79,80,81,82,83,84,85,86,87,88,89,90,91,92^) (Figure 2 and eTables 3 and 4 in Supplement 1) and included a total of 3246 participants (mean [SD] age; 37.8 [8.0] years; mean [SD] percentage female, 44.1% [17.2%]). There was a mean (SD) percentage of 31.5% (28.1%) individuals who self-reported Black race. The study populations were heterogeneous in terms of age, sex, and race, and the TMS protocols were heterogeneous in stimulation target and protocol (eTable 2 in Supplement 1). Because we only included RCTs, studies generally had low risk of bias (eTables 5 and 6 in Supplement 1).

### RCTs of TMS for OCD

**Figure 3.**
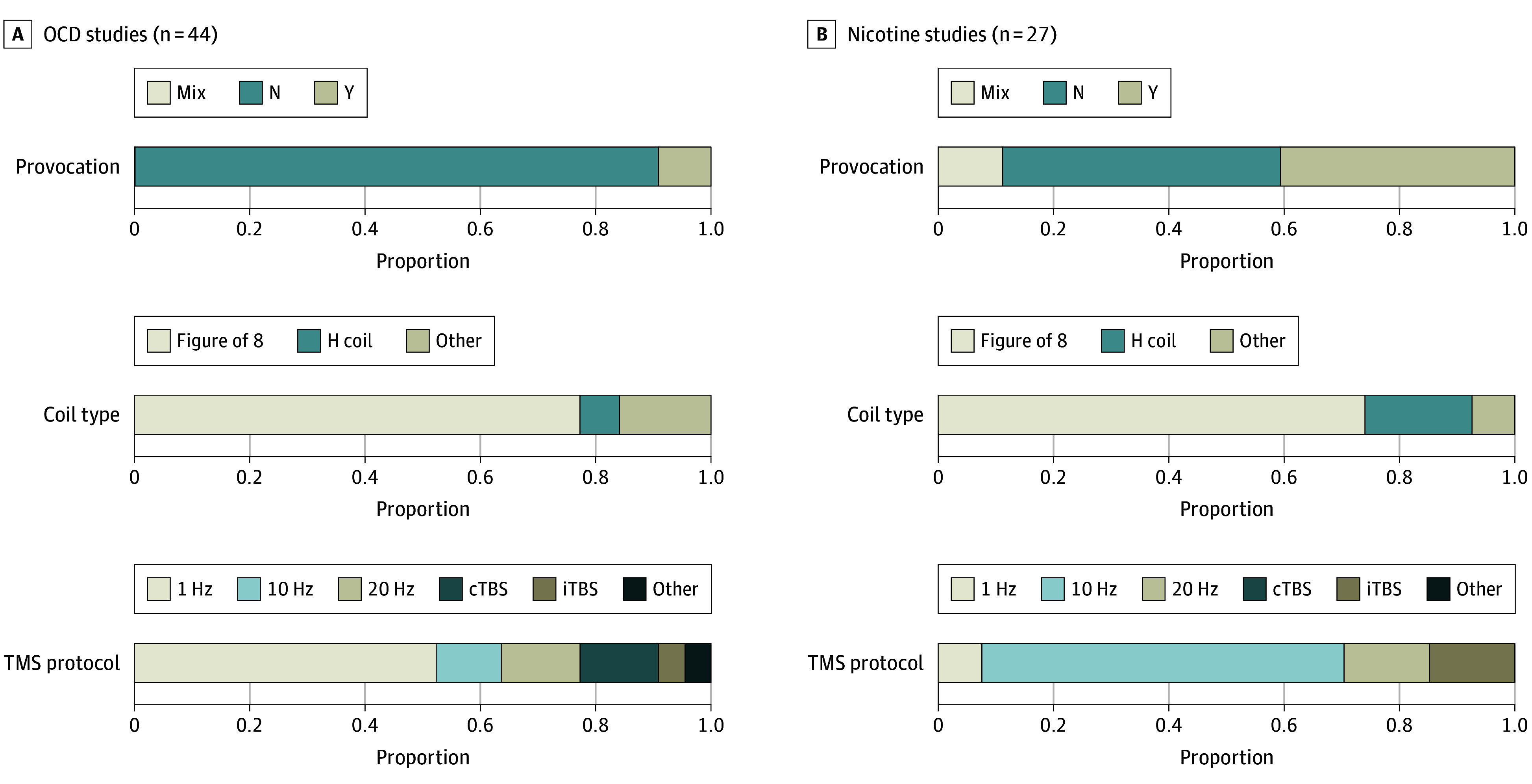
Systematic Review Results for Obsessive-Compulsive Disorder (OCD) and Nicotine Studies (N = 71) Displayed here are study characteristics (use of provocation, transcranial magnetic stimulation [TMS] coil, stimulation protocol) for studies of OCD (n = 44) (A) and nicotine dependence (n = 27) (B) included in the systematic review. The “other” category for TMS protocol includes 1 study^64^ with 6 Hz then 1 Hz and 1 study^47^ with alpha TMS: 8 to 12 Hz.

For OCD studies, baseline mean (SD) YBOCS score was 25.8 (4.3) (eTable 2 in Supplement 1). The most common TMS target was supplementary motor area (SMA) or pre-SMA (18^29,30,33,35,37,38,39,42,43,44,49,50,52,55,56,64,65,67^ of 44 studies [40.1%]), and the most commonly used TMS protocol was 1 Hz (23^28,29,30,32,33,35,39,40,42,43,44,45,49,50,51,54,56,57,59,63,65,66,67^ of 44 studies [52.3%]). Several studies compared multiple TMS targets^15,26,27,34^ and protocols^47,64^ (Figure 3 and eTables 2 and 3 in Supplement 1).

### RCTs of TMS for Nicotine Dependence

For nicotine dependence studies, baseline mean (SD) cigarettes smoked per day was 19.9 (5.8) and mean (SD) FTND score (where available) was 5.4 (1.3) (eTable 2 in Supplement 1). The most common TMS target was dorsolateral prefrontal cortex (unilateral or bilateral, 18^69,70,71,72,73,74,75,77,79,81,82,83,84,86,88,90,91,92^ of 27 studies [66.70%]), and the most commonly used TMS protocol was 10 Hz (17^16,17,69,70,71,72,73,74,75,80,85,86,87,88,89,90^ of 27 studies [63.0%]). Several studies compared multiple TMS targets^16,17,70,89^ and protocols^78,80,91^ (Figure 3; eTables 2 and 4 in the Supplement).

### Overall Study Analysis

Across all 71 studies included in the systematic review, most studies were parallel-arm RCTs (60 of 71 [84.5%]).^15,16,17,20,26,27,28,29,30,31,32,33,34,35,37,38,39,40,42,43,44,45,46,47,48,49,50,51,52,55,56,57,58,59,60,61,62,63,64,65,67,68,69,70,73,74,75,76,77,81,82,83,84,85,86,87,88,89,90,92^ Most studies used a figure of 8 coil (54^29,30,31,32,33,34,35,36,37,38,39,40,41,42,44,45,46,48,49,50,52,53,55,56,57,58,59,60,61,62,63,64,65,66,68,69,72,73,74,75,76,77,78,79,80,81,82,83,84,85,86,88,90,92^ of 71 studies [76.1%]) for multiple sessions. Eighteen studies^15,16,17,20,26,27,68,69,70,71,72,73,74,75,76,77,79,80^ included provocation with their TMS protocol, and 53 studies^28,29,30,31,32,33,34,35,36,37,38,39,40,41,42,43,44,45,46,47,48,49,50,51,52,53,54,55,56,57,58,59,60,61,62,63,64,65,66,67,78,81,82,83,84,85,86,87,88,89,90,91,92^ did not (Figure 3 and eTables 2-4 in Supplement 1). Most provocation procedures used 3 to 5 minutes of visual or auditory stimuli to provoke obsessive-compulsive symptoms or nicotine craving. Lechner et al^88^ was classified as a study that did not use provocation, as they did not use a protocol to provoke nicotine craving but rather administered a working memory task immediately before each TMS session. The eResults and eTable 7 in Supplement 1 contain provocation details.

### Meta-Analysis Results

**Figure 4.**
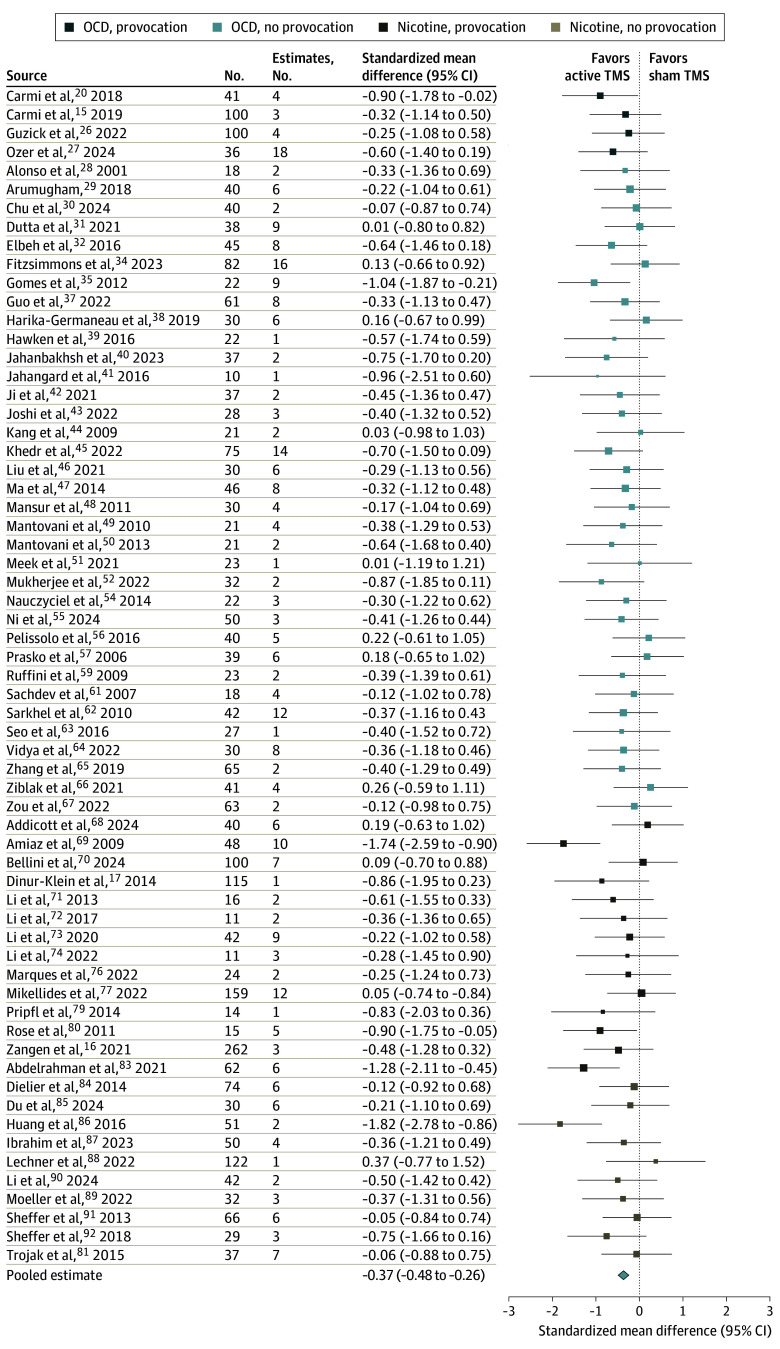
Forest Plot of Meta-Analysis Results for Obsessive-Compulsive Disorder (OCD) and Nicotine Studies Shown here are standardized mean difference and 95% CI for all studies included in meta-analysis (N = 63). Overall estimated effect size indicates reduction in symptoms, with more negative values indicating more effective treatment (pooled estimate shown with 95% CI). The effect size for each study is a weighted average of the effect sizes within each study assuming a compound symmetric correlation structure (eMethods in Supplement 1). The size of points indicates their relative weight in the meta-analysis model.

There was a total of 308 effect size estimates included in the meta-analysis from 63 studies^15,16,17,20,26,27,28,29,30,31,32,34,35,37,38,39,40,41,42,43,44,45,46,47,48,49,50,51,52,54,55,56,57,59,61,62,63,64,65,66,67,68,69,70,71,72,73,74,76,77,79,80,81,83,84,85,86,87,88,89,90,91,92^ with a total of 2998 participants (eTable 8 in Supplement 1). Three studies^53,75,82^ from the original 71 extracted were excluded due to insufficient reporting for computing SMD, and 5 studies^33,36,58,60,78^ were excluded because they did not include a sham condition (eTables 3 and 4 in Supplement 1). Studies contributed between 1 and 18 estimates (Figure 4),^15,16,17,20,26,27,28,29,30,31,32,34,35,37,38,39,40,41,42,43,44,45,46,47,48,49,50,51,52,54,55,56,57,59,61,62,63,64,65,66,67,68,69,70,71,72,73,74,76,77,79,80,81,83,84,85,86,87,88,89,90,91,92^ with a mean (SD) of 4.89 (3.77) effect sizes per study. Study-level aggregated effect size estimates are shown in Figure 4.^15,16,17,20,26,27,28,29,30,31,32,34,35,37,38,39,40,41,42,43,44,45,46,47,48,49,50,51,52,54,55,56,57,59,61,62,63,64,65,66,67,68,69,70,71,72,73,74,76,77,79,80,81,83,84,85,86,87,88,89,90,91,92^ There was a significantly better clinical response with active than sham TMS treatment (model 1 intercept, SMD [SE] = −0.37 [0.06]; 95% CI, −0.48 to −0.26) (Table), corresponding to a moderate effect size of TMS vs sham across all diagnostic groups and provocation protocols.

**Table.** Meta-Analysis Model Estimates and Multivariate Model Contrasts

Model/coefficient	Estimate (SE) [95% CI]	*P *value
**Meta-analysis model estimates**		
Model 1		
Intercept	−0.37 (0.06) [−0.48 to −0.26]	<.001
Model 2		
Intercept	−0.31 (0.06) [−0.43 to −0.18]	<.001
Provocation: yes	−0.23 (0.21) [−0.68 to 0.21]	.28
Model 3		
Intercept	−0.45 (0.12) [−0.69 to −0.21]	<.001
Group: OCD	0.14 (0.13) [−0.12 to 0.40]	.29
Model 4		
Intercept	−0.35 (0.18) [−0.74 to 0.04]	.08
Provocation: yes	−0.21 (0.36) [−1.00 to 0.58]	.57
Group: OCD	0.06 (0.19) [−0.33 to 0.45]	.75
Provocation/group interaction	−0.01 (0.39) [−0.91 to 0.89]	.98
**Multivariate model contrasts**		
Study group^a^		
OCD + provocation	−0.51 (0.14) [−0.96 to −0.07]	.04
OCD, no provocation	−0.29 (0.06) [−0.40 to −0.17]	<.001
Nicotine + provocation	−0.56 (0.25) [−1.12 to 0]	.05
Nicotine, no provocation	−0.35 (0.18) [−0.74 to 0.04]	.08
Within-group provocation^b^		
Provocation in OCD	−0.22 (0.15) [−0.65 to 0.20]	.22
Provocation in nicotine	−0.21 (0.36) [−1.00 to 0.58]	.57

Abbreviation: OCD, obsessive-compulsive disorder.

^a^ Study group compares each active treatment to sham.

^b^ Within-group provocation compares the effect sizes for the 2 active treatments.

The overall estimated benefit of symptom provocation compared with no provocation was not statistically significant (model 2 provocation, SMD [SE] = −0.23 [0.21]; 95% CI, −0.68 to 0.21; *P* = .28) (Table). There was not a significant difference in the effect size of TMS vs sham between diagnostic groups OCD and nicotine (model 3 group, SMD [SE] = 0.14 [0.13]; 95% CI, −0.12 to 0.40; *P* = .29) (Table), suggesting that TMS was similarly effective between the 2 diagnostic groups.

Model 4 included terms for diagnostic group, symptom provocation, and their interaction and did not show a significant interaction (model 4) (Table). Within this model, we compared the treatment (active vs sham) for each combination of diagnostic group and provocation setting. We found a significant treatment difference in nicotine studies with provocation (SMD [SE] = −0.56 [0.25]; 95% CI, −1.12 to 0; *P* = .05) (Table) and in OCD studies with provocation (SMD [SE] = −0.51 [0.14]; 95% CI, −0.96 to −0.07; *P* = .04) and without provocation (SMD [SE] = −0.29 [0.06]; 95% CI, −0.40 to −0.17; *P* < .001) (Table). The estimated treatment difference in nicotine studies without provocation was also negative and nonsignificant (SMD [SE] = −0.35 [0.18]; 95% CI, −0.74 to 0.04; *P* = .08) (Table).

The within-group estimates of symptom provocation (ie, the difference in effect size comparing active treatment with and without provocation) showed benefit of symptom provocation, although not statistically significant (Table). For OCD studies, the estimated effect size with provocation was 0.22 lower than without provocation (95% CI, −0.65 to 0.20; *P* = .22) (Table). In nicotine studies, the estimated effect size with provocation was 0.21 lower than without provocation (95% CI, −1.00 to 0.58; *P* = .57) (Table). These point estimates suggest a greater reduction in symptoms with symptom provocation than without provocation, according to the Cohen guidelines for effect sizes.^81^

Sensitivity analyses excluding potentially biased study findings indicated by the funnel plot yielded qualitatively similar results (eFigure and eTables 9 and 10 in Supplement 1). The random-effects analysis indicated a small amount of variance due to heterogeneity of effect sizes within studies (level 2 *I*^2^ = 7.16%) and a moderate amount due to heterogeneity between studies (level 3 *I*^2^ = 56.75%) (eResults in Supplement 1. In an exploratory analysis controlling for excitatory or inhibitory TMS protocol, the effect size for protocol was small and not significant, suggesting that excitatory or inhibitory TMS protocol was not associated with treatment response (eTable 11 in Supplement 1).

## Discussion

The current FDA-cleared TMS protocols for OCD and nicotine dependence include provocation of a symptomatic state before TMS, but the association of symptom provocation with clinical response to TMS has not been quantified. This was the first meta-analysis, to our knowledge, to quantify the association between symptom provocation and TMS clinical response. This systematic review identified 71 RCTs that tested TMS interventions for OCD or nicotine dependence. Our multilevel random-effects meta-analysis from 63 studies supported the efficacy of active over sham treatment overall for both OCD and nicotine dependence. When comparing active and sham TMS, we observed significant effects of active TMS with or without provocation for OCD and with provocation for nicotine use over sham. We then investigated the individual effect of provocation across all studies. Although we did not observe a statistically significant association of provocation with better response to TMS, there were stronger effect sizes of TMS with provocation compared with TMS without provocation for nicotine use and OCD.

To definitively determine the direct association of symptom provocation with TMS response, studies would ideally compare TMS protocols with and without provocation. Yet, only a very small minority of studies have directly compared TMS protocols with and without symptom provocation in the same sample. Two of 3 studies that directly tested provocation in the same sample^17,69^ support its use for nicotine dependence. Dinur-Klein and colleagues^17^ observed that 10-Hz TMS with provocation reduced nicotine dependence (ie, FTND) more than any other protocol tested with or without provocation. Amiaz and colleagues^69^ also observed that TMS significantly reduced cue-induced craving only when administered immediately after provocation. The results of these studies in combination with the findings from our meta-analysis suggest that symptom provocation was associated with an improved clinical response to TMS for nicotine use and OCD.

As the research and clinical applications of TMS expand, it is prudent to evaluate the effectiveness of symptom provocation for each disorder to ensure the benefit outweighs any potential risk. Additional research is warranted to more accurately estimate any added benefit of symptom provocation. If beneficial, using symptom provocation with TMS protocols would be broadly accessible and could be readily implemented in TMS clinics across the globe. However, provoking symptoms is often uncomfortable for both the patient and TMS technician.^14^ A real and unmeasured concern is that symptom provocation may simultaneously improve TMS response while also predisposing someone to drop out or relapse. Although the immediate risk of death from relapse on nicotine is near zero, the risk of death from relapse while taking opioids can be quite high. Therefore, it is worth empirically determining if symptom provocation is safe and effective before it is applied universally in TMS protocols. Symptom provocation should be used where effective but avoided when there is no clear benefit.

### Limitations

This study has some limitations. One potential limitation of our analysis was the high degree of heterogeneity between studies. The included studies were heterogeneous in terms of the TMS target, number of sessions, types of provocation, and stimulation parameters (frequency, montage, % motor threshold), but our meta-analysis did not find a significant effect of TMS protocol on response to provocation.

It is also possible that there may be protocol-dependent effects of symptom provocation. For example, when Isserles et al^19^ tested TMS for posttraumatic stress disorder, active TMS preceded by trauma script-driven imagery was more effective than both active TMS after script-driven imagery of a positive experience and sham TMS with trauma imagery. However, in another study by Isserles et al,^93^ sham TMS with trauma imagery reduced PTSD symptoms more than active TMS with trauma imagery. A key difference between the symptom provocation protocols in these 2 studies is that in Isserles et al,^19^ the trauma script (or positive script) was immediately followed by a neutral script before TMS was administered.^19^ However, in Isserles et al,^93^ the trauma script was immediately followed by TMS.^93^ This suggests that differences in provocation methods may explain differential outcomes, which should be investigated in future studies. Similarly, it is also important to consider interindividual variability in responses to drug-related cues and its impact on clinical response to TMS, which has recently been observed for depression.^94^

Finally, symptom provocation is just 1 method of inducing a particular brain state before applying TMS. There is ongoing research to test interactions between the effects of other brain state manipulations (such as oscillatory state) on TMS response.^6^

## Conclusions

In conclusion, results of this systematic review and meta-analysis to investigate the association of symptom provocation with clinical response to TMS suggest that symptom provocation may improve clinical response to TMS for nicotine use and OCD. Larger, well-powered RCTs that directly compare TMS with and without provocation are critical to establish the effect of symptom provocation on TMS response.

## Notes

Supplement 1.
eMethods.
**eTable 1.** Search Strategy
eResults.
**eTable 2.** Study and Patient Characteristics for Extracted Studies (N=71) **eTable 3.** Summary Table of Included Studies of TMS for OCD (N=44) **eTable 4.** Summary Table of Included Studies of TMS for Nicotine Dependence (N=27) **eTable 5.** Quality Assessment of Included Studies of TMS for OCD (N=44) **eTable 6.** Nicotine Dependence Quality Assessment (N=27) **eTable 7.** Provocation Protocols Used for Studies of OCD and Nicotine Dependence (N=18) **eTable 8.** Study and Patient Characteristics for Studies Included in Meta-Analysis (N=63) **eTable 9.** Sensitivity Analysis Excluding Repeated Measures ANOVA Estimates **eTable 10.** Sensitivity Analysis Excluding Potential Outliers From Funnel Plot **eTable 11.** Effect of Provocation Controlling for TMS Protocol in OCD and Nicotine Dependence **eFigure.** Funnel Plot for Meta-Analytic Model

Supplement 2.
Data Sharing Statement.
