# Supplementary material for: Symptom Provocation and Clinical Response to Transcranial Magnetic Stimulation: A Systematic Review and Meta-Analysis
Source: JAMA Psychiatry. 2025 Jun 4;82(8):768–77. doi: 10.1001/jamapsychiatry.2025.0792 (PMC12138803; doi:10.1001/jamapsychiatry.2025.0792)
Supplement: Supplement 1. — eMethods. eTable 1. Search Strategy eResults. eTable 2. Study and Patient Characteristics for Extracted Studies (N=71) eTable 3. Summary Table of Included Studies of TMS for OCD (N=44) eTable 4. Summary Table of Included Studies of TMS for Nicotine Dependence (N=27) eTable 5. Quality Assessment of Included Studies of TMS for OCD (N=44) eTable 6. Nicotine Dependence Quality Assessment (N=27) eTable 7. Provocation Protocols Used for Studies of OCD and Nicotine Dependence (N=18) eTable 8. Study and Patient Characteristics for Studies Included in Meta-Analysis (N=63) eTable 9. Sensitivity Analysis Excluding Repeated Measures ANOVA Estimates eTable 10. Sensitivity Analysis Excluding Potential Outliers From Funnel Plot eTable 11. Effect of Provocation Controlling for TMS Protocol in OCD and Nicotine Dependence eFigure. Funnel Plot for Meta-Analytic Model [file jamapsychiatry-e250792-s001.pdf]

Supplementary Online Content

Bello D, Jones M, Gadiyar I, et al. Symptom provocation and clinical response to transcranial magnetic stimulation: a systematic review and meta-analysis. *JAMA Psychiatry*. Published online June 4, 2025. doi:10.1001/jamapsychiatry.2025.0792

eMethods.

eTable 1. Search Strategy

eResults.

eTable 2. Study and Patient Characteristics for Extracted Studies (N=71)

eTable 3. Summary Table of Included Studies of TMS for OCD (N=44)

eTable 4. Summary Table of Included Studies of TMS for Nicotine Dependence (N=27)

eTable 5. Quality Assessment of Included Studies of TMS for OCD (N=44)

eTable 6. Nicotine Dependence Quality Assessment (N=27)

eTable 7. Provocation Protocols Used for Studies of OCD and Nicotine Dependence (N=18)

eTable 8. Study and Patient Characteristics for Studies Included in Meta-Analysis (N=63)

eTable 9. Sensitivity Analysis Excluding Repeated Measures ANOVA Estimates

eTable 10. Sensitivity Analysis Excluding Potential Outliers From Funnel Plot

eTable 11. Effect of Provocation Controlling for TMS Protocol in OCD and Nicotine Dependence

eFigure. Funnel Plot for Meta-Analytic Model

This supplementary material has been provided by the authors to give readers additional information about their work.

© 2025 Bello D et al. *JAMA Psychiatry*.

1. Supplementary Methods

Systematic Review Eligibility Criteria

All types of TMS were included, e.g., intermittent theta burst stimulation (iTBS), continuous theta burst stimulation (cTBS), and traditional high and low frequency TMS. Studies involving children (<18 years old) were excluded. Only randomized, controlled trials were included in this systematic review. Cohort studies, case control studies, cross-sectional studies, time-series, systematic chart reviews, letters to the editor, conference abstracts and posters, literature reviews, case reports, meta-analyses were excluded. Publications without outcome variables, such as protocols or without published quantitative data, were also excluded. Following the initial search and screen, we manually searched the references and performed a citation analysis of the included studies to identify any additional articles that met inclusion criteria. See eTable 1 for search strategy.

Meta-Analysis

For each study, the method for estimating SMD for the difference between active and sham TMS interventions was selected based on the reported statistical values. We computed Hedge's *g* and the associated standard error using the following parameters (in order of preference): the post-treatment means, standard deviations, and sample sizes; independent samples *t*-test comparing active and sham groups, and its degrees of freedom; independent *F*-test comparing active and sham groups, and its degrees of freedom; mean difference and standard error/*p*-value comparing active and sham groups; odds ratio comparing active and sham group via the formula

© 2025 Bello D et al. *JAMA Psychiatry*.

given in Sánchez-Meca 2003; pre-post change scores for active and sham groups, standard deviations, and sample sizes; F-test from repeated measures ANOVA and its degrees of freedom; t-statistic and degrees of freedom for group x time interaction; or partial η^2 for a group x time interaction. Note that for the group x time effects given by the F, t, and partial η^2 statistics, estimating the standard error required use of a formula intended for independent groups, which is unlikely to be accurate with the time component. As such, we also conduct a sensitivity analysis without these effects. Effect sizes and standard errors were calculated using functions from the metafor, esc, and effect size R packages. When creating forest plots, effect sizes and confidence intervals for each study are obtained by using a weighted average assuming a compound symmetric correlation structure. The covariance is estimated from the random effects model. Full code for the analyses is provided as supplementary material and on github (https://github.com/statimagcoll/BelloJones_Ward2024).

© 2025 Bello D et al. *JAMA Psychiatry*.

2. eTable 1. Search Strategy

PubMed

1	“Obsessive-compulsive Disorder”[Mesh]	18,142
2	“Tobacco Use Disorder”[Mesh]	12,637
3	OCD[tiab]	12,766
4	Nicotine addiction[tiab]	2,220
5	Tobacco use disorder[tiab]	626
6	Obsessive compulsive disorder[tiab]	16,644
7	Nicotine use disorder[tiab]	104
8	Nicotine dependence[tiab]	6,559
9	Tobacco addiction[tiab]	1,111
10	Tobacco dependence[tiab]	2,219
11	1 OR 2 OR 3 OR 4 OR 5 OR 6 OR 7 OR 8 OR 9 OR 10	45,095
12	“Transcranial Magnetic Stimulation”[Mesh]	16,061
13	TMS[tiab]	16,365
14	rTMS[tiab]	6,822
15	Transcranial magnetic stimulation[tiab]	20,506
16	Repetitive transcranial magnetic stimulation[tiab]	6,769
17	Theta burst stimulation[tiab]	2,276
18	iTBS[tiab]	924

© 2025 Bello D et al. *JAMA Psychiatry*.

19	Continuous theta burst stimulation[tiab]	563
20	cTBS[tiab]	889
21	Intermittent theta burst stimulation[tiab]	728
22	12 OR 13 OR 14 OR 15 OR 16 OR 17 OR 18 OR 19 OR 20 OR 21	30,914
23	"Randomized Controlled Trial"[Publication Type]	621,291
24	11 AND 22 AND 23	**61**

Embase

1	exp obsessive compulsive disorder/	53,407
2	exp tobacco dependence/	26,768
3	(nicotine dependence or nicotine addiction or tobacco dependence or tobacco addiction or OCD or obsessive compulsive disorder).ab,kf,ti.	42,713
4	exp transcranial magnetic stimulation/	34,394
5	(transcranial magnetic stimulation or TMS or rTMS or repetitive transcranial magnetic stimulation or theta burst stimulation or iTBS or intermittent theta burst stimulation or cTBS or continuous theta burst stimulation).ab,kf,ti.	41,497
6	1 OR 2 OR 3	89,826
7	4 OR 5	47,936
8	6 AND 7	1,320
9	Limit 8 to randomized controlled trial	**157**

© 2025 Bello D et al. *JAMA Psychiatry*.

PsychInfo

1	(MAINSUBJECT.EXACT.EXPLODE("Obsessive Compulsive Disorder") OR MAINSUBJECT.EXACT.EXPLODE("Tobacco Use Disorder") OR TI,AB("tobacco addiction" OR "tobacco dependence" OR "OCD" OR "obsessive compulsive disorder" OR "nicotine addiction" OR "nicotine use disorder" OR "nicotine dependence" OR "tobacco use disorder"))	30,091
2	(MAINSUBJECT.EXACT.EXPLODE("Transcranial Magnetic Stimulation") OR TI,AB("TMS" OR "transcranial magnetic stimulation" OR "rTMS" OR "repetitive transcranial magnetic stimulation" OR "theta burst stimulation" OR "iTBS" OR "intermittent theta burst stimulation" OR "cTBS" OR "continuous theta burst stimulation"))	14,377
3	1 AND 2	**330**

Cinahl

1	(MH "Obsessive-Compulsive Disorder+") OR (MH "Substance Use Disorders+") OR TI (nicotine use disorder OR nicotine addiction OR nicotine dependence OR tobacco use disorder OR tobacco addiction OR tobacco dependence OR OCD OR obsessive compulsive disorder) OR AB (nicotine use disorder OR nicotine addiction OR nicotine dependence OR tobacco use disorder OR tobacco addiction OR tobacco dependence OR OCD OR obsessive compulsive disorder)	202,450
2	(MH "Transcranial Magnetic Stimulation") OR TI (transcranial magnetic stimulation TMS OR rTMS OR	4,328

© 2025 Bello D et al. *JAMA Psychiatry*.

	repetitive transcranial magnetic stimulation OR theta burst stimulation OR iTBS OR intermittent theta burst stimulation OR cTBS OR continuous theta burst stimulation) OR AB (transcranial magnetic stimulation TMS OR rTMS OR repetitive transcranial magnetic stimulation OR theta burst stimulation OR iTBS OR intermittent theta burst stimulation OR cTBS OR continuous theta burst stimulation)	
3	1 AND 2	**182**

eTable 1. Search Strategy. Search terms and results are displayed for each of PubMed, PsychInfo, Embase, and Cinahl.

© 2025 Bello D et al. *JAMA Psychiatry*.

3. Supplementary Results

Study Characteristics

The study populations were heterogeneous in terms of age, gender, and race, and the TMS protocols were heterogeneous in terms of stimulation target and protocol (eTable 2). Seventy-one studies (n=44 OCD, n=27 nicotine dependence) comprising 3,246 participants were included in the systematic review.

For OCD studies, the most common TMS targets were supplementary motor area (SMA) or pre-SMA (18/44, 40.1%), dorsolateral prefrontal cortex (DLPFC, unilateral or bilateral, 15/44, 34.1%), frontal pole or orbitofrontal cortex (OFC, 6/44, 13.6%), and dorsomedial prefrontal cortex (DMPFC) or anterior cingulate cortex (ACC, 5/44, 11.4%). The most common TMS protocols were 1 Hz (23/44, 52.3%) followed by 10 Hz (5/44, 11.4%), cTBS (6/44, 13.6%), 20 Hz TMS (6/44, 13.6%), and iTBS (2/44, 4.5%). Several studies compared multiple TMS targets and protocols, see eTables 2-3 for details.

The most common TMS targets for nicotine dependence were DLPFC (unilateral or bilateral, 18/27, 66.7%), prefrontal and insular cortices (5/27, 18.5%), and dorsomedial prefrontal cortex and anterior cingulate cortex (DMPFC/ACC (3/27, 11.1%). The most common TMS protocols were 10 Hz (17/27, 63.0%) followed by 20 Hz (4/27, 14.8%), iTBS (4/27, 14.8%), 1 Hz (2/27, 7.4%). Several studies compared multiple TMS targets and protocols, see eTable 2 & 4 for details.

Provocation protocols were heterogeneous (eTable 7). All four studies of TMS for OCD used similar provocation protocols involving the use of a personalized hierarchy of provocations to achieve a participant-rated level of distress between 4 to 7 out of 10 on a visual analog scale. The 14 studies using provocation for nicotine dependence had greater heterogeneity in provocation protocols:

© 2025 Bello D et al. *JAMA Psychiatry*.

10/14 (71.4%) used visual cues (pictures or video), 1/14 (7.1%) used auditory cues, 4/14 (28.6%) used in vivo cues (i.e., holding a pack of cigarettes), and 5/14 (35.7%) used multiple modalities of provocation. Only 2/14 (14.3%) of nicotine dependence studies used personalized provocations.

Meta-Analysis Results

Sixty-three studies comprising 2,998 participants were included in the meta-analysis. The random effects analysis indicated a small to moderate amount of variance due to heterogeneity of effect sizes within studies ($I^2_{\text{Level 2}}$ = 7.16%) and a moderate amount due to heterogeneity between studies ($I^2_{\text{Level 3}}$ = 56.75%). While we cannot control for all factors that contribute to study heterogeneity, the variance components provide some insight into what factors are driving variability: within-study heterogeneity (7.16%) is likely due to differences in the outcome variable as other features were usually fixed within a study, and between-study heterogeneity (56.75%) can be affected by differences in outcomes as well as all features that change across studies.

There was some evidence of potential publication bias (eFigure 1), with 22 out of the 308 effect size estimates falling outside of the expected range assuming no publication bias. Sixteen of 22 estimates have a more negative than expected effect size (i.e., a stronger effect in the direction of effective active vs. sham treatment). The results of the sensitivity analyses (a) without effect size estimates from repeated measures ANOVA and (b) without outliers from the funnel plot did not change the direction of the estimated subgroup effects or statistical significance of our analyses, except for the effect for nicotine studies without provocation being much

© 2025 Bello D et al. *JAMA Psychiatry*.

more highly significant in (b) (eTable 10). The magnitude of the estimated effect size in nicotine studies with provocation was weaker in (b) than in the main analysis (-0.33 vs. -0.56, Table 2 and eTable 10).

© 2025 Bello D et al. *JAMA Psychiatry*.

4. eTable 2. Study and Patient Characteristics for Extracted Studies (N=71)

	Nicotine (N=27)	OCD (N=44)	Overall (N=71)
**Study Design**			
Crossover RCT	6 (22.2%)	5 (11.4%)	11 (15.5%)
Parallel Arm RCT	21 (77.8%)	39 (88.6%)	60 (84.5%)
**Provocation**			
Mix	3 (11.1%)	0 (0%)	3 (4.2%)
N	13 (48.1%)	40 (90.9%)	53 (74.6%)
Y	11 (40.7%)	4 (9.1%)	15 (21.1%)
**TMS Protocol**			
1 Hz	2 (7.4%)	23 (52.3%)	25 (35.2%)
10 Hz	17 (63.0%)	5 (11.4%)	22 (31.0%)
20 Hz	4 (14.8%)	6 (13.6%)	10 (14.1%)
cTBS	0 (0%)	6 (13.6%)	6 (8.5%)
iTBS	4 (14.8%)	2 (4.5%)	6 (8.5%)
Other	0 (0%)	2 (4.5%)	2 (2.8%)
**Coil Type**			
Figure of 8	20 (74.1%)	34 (77.3%)	54 (76.1%)
H coil	5 (18.5%)	3 (6.8%)	8 (11.3%)
Solid focal coil	1 (3.7%)	0 (0%)	1 (1.4%)
Butterfly double-cone	0 (0%)	1 (2.3%)	1 (1.4%)

© 2025 Bello D et al. *JAMA Psychiatry*.

Circular coil	0 (0%)	2 (4.5%)	2 (2.8%)
Double-cone	0 (0%)	2 (4.5%)	2 (2.8%)
M-100 Ultimate TMS	0 (0%)	1 (2.3%)	1 (1.4%)
MediStim (MS-30) TMS therapy system	0 (0%)	1 (2.3%)	1 (1.4%)
Missing	1 (3.7%)	0 (0%)	1 (1.4%)
**Number of Sessions**			
Mean (SD)	10.6 (8.12)	17.8 (8.95)	15.0 (9.28)
**Active TMS Target**			
DLPFC (L, R, or Bilateral)	18 (66.7%)	15 (34.1%)	33 (46.5%)
Pre-SMA/SMA	0 (0%)	18 (40.1%)	18 (25.4%)
Frontal pole/OFC	1 (3.7%)	6 (13.6%)	7 (9.9%)
DMPFC/ACC	3 (11.1%)	5 (11.4%)	8 (11.3%)
PFC and Insula	5 (18.5%)	0 (0%)	5 (7.0%)
**Neuronavigation**			
N	19 (70.4%)	34 (77.3%)	53 (74.6%)
Y	8 (29.6%)	10 (22.7%)	18 (25.4%)
**Number of Enrolled Participants**			
Mean (SD)	58.1 (55.9)	38.1 (223)	45.7 (39.5)
**Mean Age**			
Mean (SD)	43.3 (8.26)	34.6 (5.76)	37.8 (7.98)
Missing	2 (7.4%)	2 (4.5%)	4 (5.6%)

© 2025 Bello D et al. *JAMA Psychiatry*.

**Sex: Percent Female**			
Mean (SD)	36.0 (18.3)	49.0 (14.6)	44.1 (17.2)
Missing	3 (11.1%)	4 (9.1%)	7 (9.9%)
**Race: Percent Black**			
Mean (SD)	34.9 (27.9)	N/A	31.5 (28.1)
Missing	19 (70.4%)	43 (97.7%)	62 (87.3%)
**Mean Baseline YBOCS**			
Mean (SD)	N/A	25.8 (4.29)	25.8 (4.29)
Missing	27 (100%)	4 (9.1%)	31 (43.7%)
**Mean Baseline FTND**			
Mean (SD)	5.39 (1.29)	N/A	5.39 (1.29)
Missing	7 (25.9%)	44 (100%)	51 (71.8%)
**Mean Baseline Cigs/Day**			
Mean (SD)	19.9 (5.84)	N/A	19.9 (5.84)
Missing	5 (18.5%)	44 (100%)	49 (69.0%)

eTable 2. Study and Patient Characteristics for Extracted Studies (N=71). The overall characteristics are described and further classified into OCD or nicotine studies. ACC: anterior cingulate cortex; cTBS: continuous theta burst stimulation; DLPFC: dorsolateral prefrontal cortex; DMPFC: dorsomedial prefrontal cortex; iTBS: intermittent theta burst stimulation; OFC: orbitofrontal cortex; PFC: prefrontal cortex; RCT: randomized, controlled trial; SMA: supplementary motor area.

© 2025 Bello D et al. *JAMA Psychiatry*.

5. eTable 3. Summary Table of Included Studies of TMS for OCD (N=44)

Author, Year	Group	Design	Provocation?	TMS Parameters	Coil	Control	Stimulation Site	Sessions	Pulses	Outcomes
Carmi et al, 2018[1]	Treatment-resistant OCD	Parallel	Y	20 Hz, 100% RMT	H7 Coil	Sham	ACC	25	2000	YBOCS^
Carmi et al, 2019[2]	OCD	Parallel	Y	20 Hz, 100% RMT	H coil	Sham	DMPFC and ACC	29	2000	YBOCS^
Guzick et al, 2022[3]	OCD	Parallel	Y	20 Hz, 100% RMT	H coil	Sham	BL DMPFC and ACC	29	Not Reported	YBOCS^
Ozer et al, 2024[4]	OCD	Parallel	Y	20 Hz	Double-cone	Sham	mPFC and ACC	30	2000	YBOCS^, DOC, HAM-A^, HAM-S
Alonso et al, 2001[5]	Outpatients with OCD	Parallel	N	1 Hz, 110% MT	Circular	Sham	R DLPFC	18	Not Reported	YBOCS^
Arumugham et al, 2018[6]	Outpatients with OCD	Parallel	N	1 Hz, 100% RMT	Figure of 8	Sham	Pre-SMA	18	1200	YBOCS^, CGI-S, HAM-A
Chu et al, 2024[7]	Outpatients with OCD	Parallel	N	1 Hz, 100% RMT	Figure of 8	Sham	Pre-SMA	10	1200	YBOCS^, HAM-A
Dutta et al, 2021[8]	Inpatients with OCD	Parallel	N	cTBS, 80% RMT	Figure of 8	Sham	L OFC	10	1200	YBOCS, HAM-A^
Elbeh et al, 2016[9]	OCD	Parallel	N	1 Hz, 100% RMT	Figure of 8	Sham	R DLPFC	10	500	YBOCS^, HAM-A
Fitzgerald et al, 2022[10]*	Treatment-resistant OCD	Parallel	N	1 Hz, 120% RMT	Figure of 8	Active Control	R SMA	20	Not Reported	YBOCS
Fitzsimmons et al, 2024[11]	OCD	Parallel	N	10 Hz, 110%	Figure of 8	Active Control	L DLPFC and Pre-SMA	16	3000	YBOCS^
Gomes et al, 2012[12]	Outpatients with OCD	Parallel	N	1 Hz, 100% RMT	Figure of 8	Sham	Pre-SMA	10	1200	YBOCS^, HAM-A
Greenberg et al, 1997[13]*	OCD	Crossover	N	20 Hz, 80% MT	Figure of 8	Active Control	R LPFC	1	600	YBOCS^

© 2025 Bello D et al. *JAMA Psychiatry*.

Guo et al, 2022[14]	OCD	Parallel	N	cTBS, 110% RMT	Figure of 8	Sham	BL SMA	20	1,200	YBOCS, HAM-A^
Harika-Germaneau et al, 2019[15]	Treatment-resistant OCD	Parallel	N	cTBS, 70% RMT	Figure of 8	Sham	Pre-SMA	30	600	YBOCS^, BAS
Hawken et al, 2016[16]	OCD	Parallel	N	1 Hz, 110% RMT	Figure of 8	Sham	SMA	25	Not Reported	YBOCS^
Jahanbakhsh et al, 2023[17]	OCD	Parallel	N	1 Hz, unknown % MT	Figure of 8	Sham	L DLPFC	15	1200	YBOCS^
Jahangard et al, 2016[18]	OCD	Crossover	N	20 Hz, 100% RMT	Figure of 8, Sham: coil 45-90° from skull	Sham	L DLPFC	10 Active, then 10 Sham	750	YBOCS^
Ji et al, 2021[19]	OCD	Parallel	N	1 Hz, 110% RMT	Figure of 8	Sham	R Pre-SMA	14	Not Reported	YBOCS^
Joshi et al, 2022[20]	Drug-Naive OCD	Parallel	N	1 Hz, 100% RMT	MediStim (MS-30) TMS therapy system	Sham	SMA	20	1600	YBOCS^
Kang et al, 2009[21]	Treatment-resistant OCD	Parallel	N	1 Hz, 110% RMT (R PFC) or 100% RMT (SMA)	Figure of 8	Sham	R PFC and SMA	10	1200	YBOCS^
Khedr et al, 2022[22]	Outpatients with OCD	Parallel	N	1 Hz, 120% RMT	Figure of 8	Sham	R DLPFC	10	1500	YBOCS^, HAM-A
Liu et al, 2021[23]	Outpatients with OCD	Parallel	N	cTBS, 80% RMT	Figure of 8	Sham	R OFC	20	600	YBOCS^, HAM-A
Ma et al, 2014[24]	OCD	Parallel	N	Alpha TMS: 8-12 Hz, 80% MT	Circular	Sham	BL DLPFC	10	648-872	YBOCS, HAM-A

© 2025 Bello D et al. *JAMA Psychiatry*.

Mansur et al, 2011[25]	Treatment-resistant outpatients with OCD	Parallel	N	10 Hz, 110% MT	Figure of 8	Sham	R DLPFC	30	2000	YBOCS^, HAM-A
Mantovani et al, 2010[26]	Outpatients with OCD	Parallel	N	1 Hz, 100% RMT	Figure of 8	Sham	Pre-SMA	40	1200	YBOCS^, YBOCS-SR^
Mantovani et al, 2013[27]	Outpatients with OCD	Parallel	N	1 Hz, 100% RMT	Figure of 8	Sham	Pre-SMA	20	1200	YBOCS^, YBOCS-SR^
Meek et al, 2021[28]	OCD	Parallel	N	1 Hz, 120% MT	Double-cone	Sham	dACC	20	1200	YBOCS, BAI
Mukherjee et al, 2022[29]	OCD	Parallel	N	cTBS, 80% MT	Figure of 8	Sham	SMA	30	900	YBOCS^, HAM-A
Naro et al, 2019[30]*	OCD	Crossover	N	iTBS, 80% AMT	Figure of 8	Sham	L DLPFC	20	600	YBOCS^
Nauczyciel et al, 2014[31]	OCD	Crossover	N	1 Hz, 120% MT	Butterfly double-cone	Sham	R OFC	10	1200	YBOCS^
Ni et al, 2024[32]	OCD	Parallel	N	cTBS	Figure of 8	Sham	R pre-SMA	50	1800	YBOCS^
Pelissolo et al, 2016[33]	Outpatients with OCD	Parallel	N	1 Hz, 100% RMT	Figure of 8	Sham	pre-SMA	20	1500	YBOCS^, MOCI, BAS
Prasko et al, 2006[34]	OCD	Parallel	N	1 Hz, 110% MT	Figure of 8	Sham	L DLPFC	10	1800	YBOCS^, HAM-A^, BAI^
Price et al, 2021[35]*	OCD	Parallel	N	iTBS, 110% RMT	Figure of 8	Active Control	L OFC	1	1200	YBOCS
Ruffini et al, 2009[36]	OCD	Parallel	N	1 Hz, 80% RMT	Figure of 8	Sham	L OFC	15	Not Reported	YBOCS^
Sachdev et al, 2001[37]*	OCD	Parallel	N	10 Hz, 110% RMT	Figure of 8	Active Control	R DLPFC	10	Not Reported	YBOCS^, STAI
Sachdev et al, 2007[38]	OCD	Parallel	N	10 Hz, 110% MT	Figure of 8	Sham	L DLPFC	10	1500	YBOCS^, MOCI

© 2025 Bello D et al. *JAMA Psychiatry*.

Sarkhel et al, 2010[39]	OCD	Parallel	N	10 Hz, 110% MT	Figure of 8	Sham	R DLPFC	10	Not Reported	YBOCS
Seo et al, 2016[40]	OCD	Parallel	N	1 Hz, 100% RMT	Figure of 8	Sham	R DLPFC	15	1200	YBOCS^, HAM-A
Vidya et al, 2022[41]	OCD	Parallel	N	6 Hz at 80% MT then 1 Hz at 100% MT	Figure of 8	Sham	SMA	10	600 (priming), 1200 (1 Hz)	YBOCS, HAM-A
Zhang et al, 2019[42]	Outpatients with OCD	Parallel	N	1 Hz, 100% RMT	Figure of 8	Sham	Pre-SMA	20	1200	YBOCS^, HAM-A
Ziblak et al, 2021[43]	Outpatients with OCD	Crossover	N	1 Hz, 110% MT	Angled Figure of 8	Sham	R OFC	20	1000	YBOCS^, HAM-A
Zou et al, 2022[44]	Inpatients with OCD	Parallel	N	1 Hz, 100% MT	M-100 Ultimate TMS	No TMS	SMA	20	1160	HAM-A, YBOCS

eTable 3. Summary Table of Included Studies of TMS for OCD (N=44). Displayed are all included studies of TMS for OCD. *Excluded from meta-analysis; ^Primary Outcome (when specified); AMT: Active Motor Threshold; BAI: Beck Anxiety Inventory; BAS: Beck Anxiety Rating Scale; BL: Bilateral; CGI-S: Clinical Global Impression-Severity; dACC: Dorsal Anterior Cingulate Cortex; DLPFC: Dorsolateral Prefrontal Cortex; DMPFC: Dorsomedial Prefrontal Cortex; HAM-A: Hamilton Anxiety Rating Scale; L: Left; MOCI: Maudsley Obsessive Compulsive Inventory; MT: Motor Threshold; OCD: Obsessive-Compulsive Disorder; OFC: Orbito-Frontal Cortex; R: Right; RMT: Resting Motor Threshold; SMA: Supplementary Motor Area; STAI: State-Trait Anxiety Inventory; TMS: Transcranial Magnetic Stimulation; YBOCS: Yale-Brown Obsessive Compulsive Scale

© 2025 Bello D et al. *JAMA Psychiatry*.

6. eTable 4. Summary Table of Included Studies of TMS for Nicotine Dependence (N=27)

Author, Year	Group	Design	Provocation	TMS Parameters	Coil	Control	Stimulation Site	Sessions	Pulses	Outcomes
Addicott et al, 2024[45]	Treatment-seeking smokers	Parallel	Y	iTBS, 110% RMT	Figure of 8	Sham	MPFC	28	600	Cig/day^, urine cotinine^, craving
Amiaz et al, 2009[46]	Treatment-Seeking Smokers	Parallel	Y	10 Hz, 100% MT	Figure of 8	Sham	L DLPFC	10	50	Cot/Cre, Cig/day, FTND, TCQ, VAS
Bellini et al, 2024[47]	Treatment-seeking smokers	Parallel	Y	10 Hz, 120% RMT	H4 coil	Sham	LPFC and BL Insula	21	1800	Cig/day, CO, cotinine, abstinence^, TCQ
Dinur-Klein et al, 2014[48]	Smokers	Parallel	Y	10 Hz, 120% RMT	H coil	Active Control	BL Insula and PFC	13	990	Cig/day^, Cot/Cre, TCQ, FTND
Li et al, 2013[49]	Non-treatment seeking, Nicotine-dependent	Crossover	Y	10 Hz, 100% RMT	Solid Focal	Sham	DLPFC	1	3000	Craving, Cue reactivity^
Li et al, 2017[50]	Non-treatment seeking Smokers	Crossover	Y	10 Hz, 100% RMT	Figure of 8	Sham	L DLPFC	1	3000	Craving^
Li et al, 2020[51]	Treatment-seeking Smokers	Parallel	Y	10 Hz, 100% RMT	Figure of 8	Sham	L DLPFC	10	3000	Cigs/day^, Cotinine, Expired CO, FTND, QSU, MNWS, Cig/day
Li et al, 2022[52]	Treatment-seeking Smokers with cancer	Parallel	Y	10 Hz, 100% RMT	Figure of 8	Sham	L DLPFC	5	3000	Cig/day^, QSU, Expired CO
Li et al, 2024[53]	Tobacco Use Disorder	Parallel	Y	10 Hz	Figure of 8	Sham	L DLPFC	5	3000	FTND, Craving^

© 2025 Bello D et al. *JAMA Psychiatry*.

Marques et al, 2022[54]	Nicotine Dependent	Parallel	Y	1 Hz, 110% RMT	Figure of 8	Active Control	L Frontal Pole	1	1200	Cue Reactivity^
Mikellides et al, 2022[55]	Treatment-seeking Smokers	Parallel	Y	iTBS, 100% RMT	Figure of 8	Sham	L DLPFC	20	600	Cig/day^, Expired CO^, FTND^, Craving^
Pripfl et al, 2014[56]	Tobacco dependent	Crossover	Y	10 Hz, 90% RMT	Figure of 8	Sham	L DLPFC	1	1200	Craving^
Rose et al, 2011[57]	Smokers	Crossover	Y	10 Hz, 1 Hz, 90% RMT	Figure of 8	Active Control	SFG	1	N/A	Craving^
Zangen et al, 2021[58]	Treatment-seeking Smokers	Parallel	Y	10 Hz, 120% MT	H4 coil	Sham	BL Insula and PFC	18	1800	Quit rate^, Cig/day, TCQ
Abdelrahman et al, 2021[59]	Male, Treatment-Seeking Smokers	Parallel	N	20 Hz, 80% RMT	Figure of 8	Sham	L DLPFC	10	2000	TCQ^, FTND^, Cig/day^
Dieler et al, 2014[60]	Smokers	Parallel	N	iTBS, 80% MT	Figure of 8	Sham	R DLPFC	4	600	Abstinence^, SER, QSU
Du et al, 2024[61]	Smokers with Schizophrenia	Parallel	N	10 Hz, electric field strength > 100 V/m	Figure of 8	Sham	DMPFC	20	1200	Cig/day, FTND^, cotinine
Huang et al, 2016[62]	Male smokers with Schizophrenia	Parallel	N	10 Hz	Figure of 8	Sham	L DLPFC	21	2000	Cig/day^
Ibrahim et al, 2023[63]	Nicotine Dependent	Parallel	N	10 Hz	H11-coil	Sham	Insula	20	1020	FTND, Craving, cig/day, CO
Lechner et al, 2022[64]	Nicotine Dependent	Parallel	N	10 Hz + WMT, 100% RMT	Figure of 8	Sham	L DLPFC	10	2000	Time to Lapse^, Cig/day
X Li et al, 2024[65]	Treatment-seeking Smokers	Parallel	N	10 Hz	Figure of 8	Sham	L DLPFC	10	3000	% change in cig use^, quit rate
Moeller et al, 2022[66]	Smokers with Schizophrenia	Parallel	N	10 Hz, 120% RMT	H4 coil	Sham	Insula and PFC	15	N/A	Cig smoked, self-admin^

© 2025 Bello D et al. *JAMA Psychiatry*.

Newman-Norlund et al, 2020[67]*	Smokers	Crossover	N	iTBS, cTBS, 80% RMT	Figure of 8	Active Control	R IFG	1	200	Inhibitory Control^
Sheffer et al, 2013[68]	Non-treatment seeking Smokers	Crossover	N	20 Hz, 10 Hz, 110% RMT	Not Reported	Sham	L DLPFC	1	900	Delay Discounting^, Cig/day
Sheffer et al, 2018[69]	Nicotine Dependent	Parallel	N	20 Hz, 110% RMT	Figure of 8	Sham	L DLPFC	8	900	Delay Discounting^, Relapse^
Trojak et al, 2015[70]*	Nicotine Dependent	Parallel	N	1 Hz, 120% RMT	Figure of 8	Sham	R DLPFC	10	360	Relapse^, VAS, QSU, FTCQ
Wing et al, 2012[71]*	Smokers with Schizophrenia	Parallel	N	20 Hz	Figure of 8	Sham	BL DLPFC	20	1500	QSU

eTable 4. Summary Table of Included Studies of TMS for Nicotine Dependence (N=27). Displayed are all included studies of TMS for nicotine dependence. Lechner at al., 2022[64] did not use a symptom provocation protocol but did administer a working memory task immediately before each TMS session, which has implications for the state-dependent effects of TMS.
*Excluded from meta-analysis; ^Primary Outcome (when specified); AMT: Active Motor Threshold; BL: Bilateral; CO: Carbon Monoxide; Cot/Cre: Cotinine/Creatinine Ratio; DLPFC: Dorsolateral Prefrontal Cortex; Cig/day: Cigarettes smoked per day; FTND: Fagerstrom Test for Nicotine Dependence; L: Left; MNWS: Minnesota Nicotine Withdrawal Scale; MT: Motor Threshold; QSU: Tiffany Brief Questionnaire of Smoking Urges; R: Right; RMT: Resting Motor Threshold; TCQ: Tobacco Craving Questionnaire; TMS: Transcranial Magnetic Stimulation.

© 2025 Bello D et al. *JAMA Psychiatry*.

7. eTable 5. Quality Assessment of Included Studies of TMS for OCD (N=44)

Author, Year	Sequence Generation	Allocation Concealment	Blinding of Participants and Personnel	Blinding of Outcome Assessment	Incomplete Outcome Data	Selective Reporting	Other Sources of Bias
Alonso et al, 2001[5]	Low	Low	Low	Low	Low	Low	Low
Arumugham et al, 2018[6]	Low	Low	Low	Low	Low	Low	Low
Carmi et al, 2018[1]	Low	Low	Low	Low	Low	Low	Low
Carmi et al, 2019[2]	Low	Low	Low	Low	Low	Low	Low
Chu et al, 2024[7]	Low	Low	Low	Low	Low	Low	Low
Dutta et al, 2021[8]	Low	Low	Low	Low	Low	Low	Low
Elbeh et al, 2016[9]	Low	Low	Low	Low	Low	Low	Low
Fitzgerald et al, 2022[10]	Low	Low	Low	Low	Low	Low	Low
Fitzsimmons et al, 2024[11]	Low	Low	Low	Low	Low	Low	Low
Gomes et al, 2012[12]	Low	Low	Low	Low	Low	Low	Low
Greenberg et al, 1997[13]	Low	Low	Low	Low	Low	Low	Low
Guo et al, 2022[14]	Low	Low	Low	Low	Low	Low	Low
Guzick et al, 2022[3]	Low	Low	Low	Low	Low	Low	High
Harika-Germaneau et al, 2019[15]	Low	Low	Low	Low	Low	Low	Low
Hawken et al, 2016[16]	Low	Low	Low	Low	Low	Low	Low
Jahanbakhsh et al, 2023[17]	Low	Low	Low	Low	Low	Low	Low
Jahangard et al, 2016[18]	Low	Low	Low	Low	Low	Low	Low
Ji et al, 2021[19]	Low	Low	Low	Low	Low	Low	Low
Joshi et al, 2022[20]	Low	Low	Low	Low	Low	Low	Low
Kang et al, 2009[21]	Low	Low	Low	Low	Low	Low	Low
Khedr et al, 2022[22]	Low	Low	Low	Low	Low	Low	Low
Liu et al, 2021[23]	Low	Low	Low	Low	Low	Low	Low
Ma et al, 2014[24]	Low	Low	Low	Low	Low	Low	Low
Mansur et al, 2011[25]	Low	Low	Low	Low	Low	Low	Low
Mantovani et al, 2010[26]	Low	Low	Low	Low	Low	Low	Low
Mantovani et al, 2013[27]	Low	Low	Low	Low	Low	Low	Low
Meek et al, 2021[28]	Low	Low	Low	Low	Low	Low	Low
Mukherjee et al, 2022[29]	Low	Low	Low	Low	Low	Low	Low
Naro et al, 2019[30]	Low	Low	Low	Low	Low	Low	Low
Nauczyciel et al, 2014[31]	Low	Low	Low	Low	Low	Low	Low

© 2025 Bello D et al. *JAMA Psychiatry*.

Ni et al, 2024[32]	Low	Low	Low	Low	Low	Low	Low
Ozer at al, 2024[4]	Low	Low	Low	Low	Low	Low	Low
Pelissolo et al, 2016[33]	Low	Low	Low	Low	Low	Low	Low
Prasko et al, 2006[34]	Low	Low	Low	Low	Low	Low	Low
Price et al, 2021[35]	Low	Low	Low	Low	Low	Low	Low
Ruffini et al, 2009[36]	Low	Low	Low	Low	Low	Low	Low
Sachdev et al, 2001[37]	Low	Low	Low	Low	Low	Low	Low
Sachdev et al, 2007[38]	Low	Low	Low	Low	Low	Low	Low
Sarkhel et al, 2010[39]	Low	Low	Low	Low	Low	Low	Low
Seo et al, 2016[40]	Low	Low	Low	Low	Low	Low	Low
Vidya et al, 2022[41]	Low	Low	Low	Low	Low	Low	Low
Zhang et al, 2019[42]	Low	Low	Low	Low	Low	Low	Low
Ziblak et al, 2021[43]	Low	Low	Low	Low	Low	Low	Low
Zou et al, 2022[44]	Low	Low	High	Unsure/Not enough data	Low	Low	Low

eTable 5. Quality Assessment of Included Studies of TMS for OCD (N=44). This table summarizes the Cochrane Risk of Bias quality assessment of each study of TMS for OCD included in the systematic review.

© 2025 Bello D et al. *JAMA Psychiatry*.

8. eTable 6. Nicotine Dependence Quality Assessment (N=27)

Study	Sequence Generation	Allocation Concealment	Blinding of Participants and Personnel	Blinding of Outcome Assessment	Incomplete Outcome Data	Selective Reporting	Other Sources of Bias
Abdelrahman et al, 2021[59]	Low	Low	Low	Low	Low	Low	Low
Addicott et al, 2024[45]	Low	Low	Low	Low	Low	Low	High
Amiaz et al, 2009[46]	Low	Low	Low	Low	Low	Low	Low
Bellini et al, 2024[47]	Low	Low	Low	Low	Low	Low	Low
Dieler et al, 2014[60]	Low	Low	Low	Low	Low	Low	Low
Dinur-Klein et al, 2014[48]	Low	Low	Low	Low	Low	Low	High
Du et al, 2024[61]	Low	Low	Low	Low	Low	Low	Low
Huang et al, 2016[62]	Low	Low	Low	Low	Low	Low	Low
Ibrahim et al, 2023[63]	Low	Low	Low	Low	Low	Low	High
Lechner et al, 2022[64]	Low	High	Low	Low	Low	Low	Low
Li et al, 2013[49]	Low	Low	Low	Low	Low	Low	Low
Li et al, 2017[50]	Low	Low	Low	Low	Low	Low	Low
Li et al, 2020[51]	Low	Low	Low	Low	Low	Low	Low
Li et al, 2022[52]	Low	Low	Low	Low	Low	Low	Low
Li et al, 2024[65]	Low	Low	Low	Low	Low	Low	Low
Li et al, 2024[53]	Low	Low	Low	Low	Low	Low	Low
Marques et al, 2022[54]	Low	Low	Low	Low	Low	Low	Low
Mikellides et al, 2022[55]	Low	Low	Low	Low	Low	Low	Low
Moeller et al, 2022[66]	Low	Low	Low	Low	Low	Low	Low
Newman-Norlund et al, 2020[67]	Low	Low	Low	Low	Low	Low	Low
Pripfl et al, 2014[56]	Low	Low	Low	Low	Low	Low	High
Rose et al, 2011[57]	Low	Low	Low	Low	Low	Low	Low
Sheffer et al, 2013[68]	Low	Low	Low	Low	High	Low	Low
Sheffer et al, 2018[69]	Low	Low	Low	Low	Low	Low	Low
Trojak et al, 2015[70]	Low	Low	Low	Low	Low	Low	Low
Wing et al, 2012[71]	Low	Low	Low	Low	Low	Low	Low
Zangen et al, 2021[58]	Low	Low	Low	Low	Low	Low	Low

eTable 6. Nicotine Dependence Quality Assessment (N=27). This table summarizes the Cochrane Risk of Bias quality assessment of each nicotine dependence study included in the systematic review.

© 2025 Bello D et al. *JAMA Psychiatry*.

9. eTable 7. Provocation Protocols Used for Studies of OCD and Nicotine Dependence (N=18)

Author, Year	Provocation Protocol
**OCD (N=4)**	
Carmi et al, 2018[1]	Personalized provocations designed by a clinician during first assessment designed to achieve self-reported distress between 4 to 7 on a 1-10 VAS. Did not specify visual or auditory.
Carmi et al, 2019[2]	Three to five minute personalized symptom provocation using hierarchically-ordered list of provocations from least to most distressing designed by a clinician together with the participant during first assessment designed to achieve self-reported distress between 4 to 7 on a 1-10 VAS. Administered by certified and trained staff member. Participant was instructed to continue thinking about obsessions during TMS session and was reminded about the provocation during the session.
Guzick et al, 2022[3]	Three to five minute personalized symptom provocation using hierarchically-ordered list of provocations organized from least to most distressing designed by clinician and participant during first assessment to achieve self-reported distress of 4 to 7 on a 1-10 VAS. Participant was instructed to continue thinking about obsessions during TMS session and was reminded about the provocation during the session. Both internal (questions designed to evoke obsessive doubts) and external (symptom-relevant stimuli) provocations were developed. External stimuli were used only if provocations failed to evoke sufficient distress.
Ozer et al, 2024[4]	Personalized provocations designed to achieve a self-reported distress level of 4-7 were used to provoke symptoms during TMS treatment. The questions selected for provocation were administered in order from least distressing to more distressing.
**Nicotine Dependence (N=14)**	
Addicott et al, 2024[45]	Participants were instructed to recollect their most recent smoking experience and given motivation to quit smoking by a study staff member.
Amiaz et al, 2009[46]	Participants were randomized to receive either smoking-related or neutral pictures prior to daily TMS intervention. Smoking pictures showed smoking-related activities. Neutral pictures showed non-smoking related activities. TMS session was performed immediately after provocation.
Bellini et al, 2024[47]	A 5-minute provocation procedure was given to participants, but no details were provided.
Dinur-Klein et al, 2014[48]	Participants were randomized to provocation or non-provocation groups. An in vivo smoking cue was used where another person lit up a cigarette and took one puff one meter away from the participant.
Li et al, 2013[49]	Participants were shown 70 scenic images, 40 neutral images, and 40 cigarette smoking images presented in 4 blocks as follows: 1) scenic (5 min); 2) neutral (1.5 min); 3) scenic (5 min); 4) cigarette smoking images (1.5 min)

© 2025 Bello D et al. *JAMA Psychiatry*.

Li et al, 2017[50]	Smoking-related cues were presented during TMS session. Cues consisted of 1) a house-made smoking video of a person lighting up and smoking a cigarette and 2) a series of smoking-related images of heads and mouths of people smoking, hands holding a cigarette, or cigarettes in an ashtray or pack.
Li et al, 2020[51]	In vivo exposure for 1.5 min interacting with smoking paraphernalia (cigarettes, ashtray, lighter) before each TMS session. While TMS was administered, participants watched 15-min video of people smoking in various environments.
Li et al, 2022[52]	In vivo exposure for 1.5 min interacting with smoking paraphernalia (cigarettes, ashtray, lighter) before each TMS session. While TMS was administered, participants watched 15-min video of people smoking in various environments.
Li et al, 2024[53]	Subjects performed a cue-craving task during the fMRI scan. In the task, subjects were shown 30 smoking (e.g., person smoking in front of a computer) and 30 neutral images (typing-related) for 15 minutes.
Marques et al, 2022[54]	Participants view 20 smoking-related and 20 neutral images to assess craving prior to TMS. Five additional pictures were selected for higher reactivity values and were presented separately immediately before TMS for craving induction.
Mikellides et al, 2022[55]	During TMS intervention, participants were instructed to watch two 3-min videos: 1) smoking-related video designed to elicit craving and 2) neutral video
Pripfl et al, 2014[56]	Prior to TMS, participants viewed 6 blocks of smoking images (e.g., hands holding lit cigarettes), neutral images, and a fixation cross. Block order was randomized, and blocks of the same category were not consecutive.
Rose et al, 2011[57]	During TMS administration, participants were exposed to either neutral or smoking cues. Smoking cues included holding a cigarette and a lighter while viewing a lit cigarette or smoking a cigarette via a controlled puff volume apparatus. Neutral cues consisted of participants holding a pencil and rubber eraser rather than a cigarette and lighter.
Zangen et al, 2021[58]	Participants completed 5 min provocation procedure prior to each TMS session where they 1) imagined their greatest trigger for craving; 2) listened to an audio script with instructions to handle a cigarette and lighter; and 3) viewed smoking-related pictures.

eTable 7. Description of Provocation Protocols. We describe the individual symptom provocation protocols used for each included TMS study for OCD (n=4) and nicotine dependence (n=14). Lechner at al 2022[64] did not use a symptom provocation protocol but did administer a working memory task immediately before each TMS session, which has implications for the state-dependent effects of TMS. VAS = visual analog scale.

© 2025 Bello D et al. *JAMA Psychiatry*.

10. eTable 8. Study and Patient Characteristics for Studies Included in Meta-Analysis (N=63)

	Nicotine (N=24)	OCD (N=39)	Overall (N=63)
**Study Design**			
Crossover RCT	5 (20.8%)	3 (7.7%)	8 (12.7%)
Parallel Arm RCT	19 (79.2%)	36 (92.3%)	55 (87.3%)
**Provocation**			
Mix	2 (8.3%)	0 (0%)	2 (3.2%)
N	11 (45.8%)	35 (89.7%)	46 (73.0%)
Y	11 (45.8%)	4 (10.3%)	15 (23.8%)
**Coil Type**			
Figure of 8	17 (70.8%)	29 (74.4%)	46 (73.0%)
H coil	5 (20.8%)	3 (7.7%)	8 (12.7%)
Solid focal coil	1 (4.2%)	0 (0%)	1 (1.6%)
Butterfly double-cone	0 (0%)	1 (2.6%)	1 (1.6%)
Double-cone	0 (0%)	2 (5.1%)	2 (3.2%)
Circular coil	0 (0%)	2 (5.1%)	2 (3.2%)
M-100 Ultimate TMS	0 (0%)	1 (2.6%)	1 (1.6%)

© 2025 Bello D et al. *JAMA Psychiatry*.

MediStim (MS-30) TMS therapy system	0 (0%)	1 (2.6%)	1 (1.6%)
Missing	1 (4.2%)	1 (2.6%)	2 (3.2%)
**Number of Sessions**			
Mean (SD)	10.8 (8.08)	18.7 (8.55)	15.7 (9.16)
**Active TMS Target**			
DLPFC (L, R, or Bilateral)	15 (62.5%)	12 (30.8%)	27 (42.9%)
Pre-SMA/SMA	0 (0%)	17 (43.6%)	17 (27.0%)
Frontal pole/OFC	1 (4.2%)	5 (12.8%)	6 (9.5%)
DMPFC/ACC	3 (12.5%)	5 (12.8%)	8 (12.7%)
PFC and Insula	5 (12.5%)	0 (0%)	5 (7.9%)
**Neuronavigation**			
N	17 (70.8%)	30 (76.9%)	47 (74.6%)
Y	7 (29.2%)	9 (23.1%)	16 (25.4%)
**Number of Enrolled Participants**			
Mean (SD)	60.5 (57.5)	39.6 (21.3)	47.6 (40.1)
**Mean Age**			

© 2025 Bello D et al. *JAMA Psychiatry*.

Mean (SD)	44.7 (6.90)	34.0 (5.15)	38.6 (35.7)
Missing	1 (4.2%)	1 (2.6%)	2 (3.2%)
**Sex: Percent Female**			
Mean (SD)	38.2 (17.1)	49.0 (14.0)	44.9 (16.0)
Missing	2 (8.3%)	2 (5.1%)	4 (6.3%)
**Race: Percent Black**			
Mean (SD)	35.0 (27.8)	N/A	31.4 (29.6)
Missing	16 (66.7%)	38 (97.4%)	54 (85.7%)
**Mean Baseline YBOCS**			
Mean (SD)	NA	25.6 (4.24)	25.6 (4.24)
Missing	24 (100%)	4 (10.3%)	28 (44.4%)
**Mean Baseline FTND**			
Mean (SD)	5.42 (1.35)	N/A	5.42 (1.35)
Missing	6 (25.0%)	39 (100%)	45 (71.4%)
**Mean Baseline Cigs/Day**			
Mean (SD)	20.1 (5.93)	N/A	20.1 (5.93)
Missing	3 (12.5%)	39 (100%)	42 (66.7%)

© 2025 Bello D et al. *JAMA Psychiatry*.

eTable 8. Study and Patient Characteristics for 63 Studies Included in Meta-Analysis. The overall characteristics are described and further classified into OCD or nicotine studies. ACC: anterior cingulate cortex; DLPFC: dorsolateral prefrontal cortex; OFC: orbitofrontal cortex; PFC: prefrontal cortex; RCT: randomized, controlled trial; SMA: supplementary motor area.

© 2025 Bello D et al. *JAMA Psychiatry*.

11. eTable 9. Sensitivity Analysis Excluding Repeated Measures ANOVA Estimates

		**Estimate**	**Std. Error**	**95% CI**	***p***
**Univariate Models**	*Intercept-Only Model*				
	Intercept	-0.4167	0.0656	(-0.5483, -0.2852)	<0.001
	Provocation Model				
	Intercept	-0.3467	0.0807	(-0.5099, -0.1834)	<0.001
	Provocation - Yes	-0.2581	0.2583	(-0.7970, 0.2809)	0.3297
	Group Model				
	Intercept	-0.5428	0.1376	(-0.8294, -0.2563)	<0.001
	Group - OCD	0.2060	0.1511	(-0.0987, 0.5106)	0.1800
**Multivariate Model**					
	Intercept	-0.4438	0.2391	(-0.9827, 0.0952)	0.0956
	Provocation - Yes	-0.1996	0.4612	(-1.2359, 0.8367)	0.6749
	Group - OCD	0.1345	0.2484	(-0.3894, 0.6584)	0.5952
	Provocation/Group Interaction	-0.0255	0.4873	(-1.1417, 1.0907)	0.9596
**Multivariate Model Contrasts**					
	OCD + Provocation	-0.5343	0.1421	(-0.9873, -0.0813)	0.0331
	OCD, No Provocation	-0.3093	0.0673	(-0.4470, -0.1715)	0.0001
	Nicotine + Provocation	-0.6434	0.3000	(-1.3030, 0.0162)	0.0550

© 2025 Bello D et al. *JAMA Psychiatry*.

	Nicotine, No Provocation	-0.4438	0.2391	(-0.9827, 0.0952)	0.0956
	Provocation Effect in OCD	-0.2250	0.1573	(-0.6652, 0.2151)	0.2271
	Provocation Effect in Nicotine	-0.1996	0.4612	(-1.2359, 0.8367)	0.6749

eTable 9. Sensitivity Analysis Excluding Repeated Measures ANOVA Estimates. This table describes the coefficient estimates, standard errors, 95% confidence intervals, and *p*-values for the four meta-analytic models fit in the sensitivity analysis that excluded effect size estimates from *F*-tests, *t*-tests, and partial η^2 from repeated measures ANOVA.

The results of the sensitivity analysis which excludes effect size estimates derived from repeated measures ANOVA group x time interactions (66 out of the 308 original estimates) displayed in eTable 8 are similar to that of the main model (Table 2).

© 2025 Bello D et al. *JAMA Psychiatry*.

12. eTable 10. Sensitivity Analysis Excluding Potential Outliers from Funnel Plot

		**Estimate**	**Std. Error**	**95% CI**	***p***
**Univariate Models**	*Intercept-Only Model*				
	Intercept	-0.3046	0.0411	(-0.3871, -0.2222)	<0.001
	Provocation Model				
	Intercept	-0.2777	0.0470	(-0.3726, -0.1827)	<0.001
	Provocation - Yes	-0.1021	0.0974	(-0.3035, 0.0992)	0.3051
	Group Model				
	Intercept	-0.3167	0.0675	(-0.4579, -0.1756)	<0.001
	Group - OCD	0.0183	0.0856	(-0.1546, 0.1913)	0.8315
**Multivariate Model**					
	Intercept	-0.3055	0.0903	(-0.5095, -0.1016)	0.0080
	Provocation - Yes	-0.0214	0.1385	(-0.3122, 0.2693)	0.8787
	Group - OCD	0.0365	0.1060	(-0.1891, 0.2621)	0.7353
	Provocation/Group Interaction	-0.2122	0.1992	(-0.1891, 0.2276)	0.3101
**Multivariate Model Contrasts**					
	OCD + Provocation	-0.5027	0.1319	(-0.9276, -0.0777)	0.0330
	OCD, No Provocation	-0.2690	0.0555	(-0.3824, -0.1556)	<0.001
	Nicotine + Provocation	-0.3270	0.1050	(-0.5641, -0.0898)	0.0123

© 2025 Bello D et al. *JAMA Psychiatry*.

	Nicotine, No Provocation	-0.3055	0.0903	(-0.5095, -0.1016)	0.0080
	Provocation Effect in OCD	-0.2337	0.1431	(-0.6380, 0.1707)	0.1810
	Provocation Effect in Nicotine	-0.0214	0.1385	(-0.3122, 0.2693)	0.8787

eTable 10. Sensitivity Analysis Excluding Potential Outliers from Funnel Plot. This table describes the coefficient estimates, standard errors, 95% confidence intervals, and *p*-values for the four meta-analytic models fit in the sensitivity analysis that excluded effect size estimates indicated as potential outliers from the original multivariate model.

The results of the sensitivity analysis which excludes effect size estimates indicated as potentially representative of publication bias from the funnel plot of the main multivariate model (22 out of the 308 original estimates) are displayed in eTable 9. The study group estimates are similar to the main analysis (Table 2) for OCD studies with or without provocation and nicotine studies without provocation, with the effect in nicotine studies without provocation being much more statistically significant. The effect in nicotine studies with provocation is still significant, but weaker in the sensitivity analysis. The estimated effect of provocation in nicotine studies is near 0 in the sensitivity analysis. Several of the excluded effect size estimates in this analysis were from the Amiaz 2009 paper, which used a mix of provocation and non-provocation, and effects with provocation were estimated to be greater.[41]

© 2025 Bello D et al. *JAMA Psychiatry*.

13. eTable 11. Effect of Provocation Controlling for TMS Protocol in OCD and Nicotine Dependence

	Estimate	Std. Error	95% CI	*p*
**OCD + Provocation**	-0.5108	0.1397	(-0.9569, -0.0647)	0.0357
**OCD, No Provocation**	-0.3038	0.1055	(-0.5557, -0.0518)	0.0249
**Nicotine + Provocation**	-0.5691	0.2601	(-1.1397, 0.0015)	0.0505
**Nicotine, No Provocation**	-0.3429	0.1821	(-0.7451, 0.0593)	0.0872
**Effect Size Estimates by Group and Provocation Controlling for Protocol**				
	**Estimate**	**Std. Error**	**95% CI**	***p***
**Provocation Effect in OCD Studies**	-0.2070	0.1751	(-0.6274, 0.2134)	0.2784
**Provocation Effect in Nicotine Studies**	-0.2262	0.3713	(-1.0357, 0.5833)	0.5537

eTable 11. Effect of Provocation Controlling for TMS Protocol in OCD and Nicotine Dependence. Std. Error=standard error; CI=confidence interval. Meaningful model contrasts calculated from the multivariate meta-analytic model are presented with estimates, standard errors, 95% confidence intervals, and *p*-values. This table describes the coefficient estimates, standard errors, 95% confidence intervals, and *p*-values for an exploratory analysis investigating the difference between excitatory and inhibitory TMS. In this analysis, we added a protocol covariate to Model 4 (Table 2). Excitatory sequences were defined as any TMS>1Hz or iTBS, and inhibitory sequences were defined as any TMS of 1Hz or less or cTBS. One study was removed from this analysis because it used a combination of both stimulation protocols in the active condition.[41] In this analysis, the effect size for protocol was small and not significant, suggesting that excitatory or inhibitory TMS protocol did not affect treatment response.

© 2025 Bello D et al. *JAMA Psychiatry*.

14. eFigure 1. Funnel Plot for Meta-Analytic Model

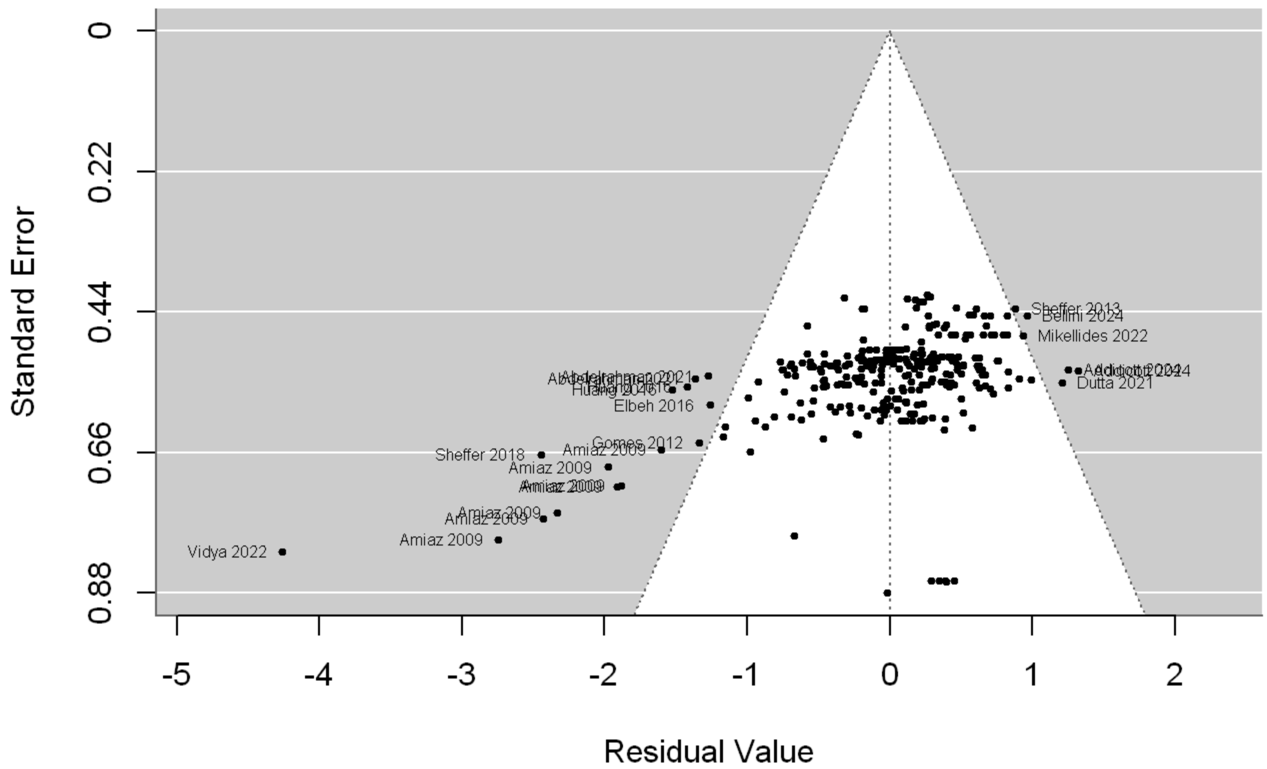

eFigure 1. Funnel Plot for Meta-Analytic Model. Under no publication bias, all estimated residuals are expected to fall within the white funnel shape. Each point represents one effect size estimate from a given study. Outliers to the left of the funnel have a stronger than expected treatment effect, and outliers to the right have a weaker than expected effect. We performed a sensitivity analysis removing estimates outside of the funnel (see eTable 9).

© 2025 Bello D et al. *JAMA Psychiatry*.

References

1. Carmi L, Alyagon U, Barnea-Ygael N, Zohar J, Dar R, Zangen A. Clinical and electrophysiological outcomes of deep TMS over the medial prefrontal and anterior cingulate cortices in OCD patients. *Brain Stimulation*. 2018;11(1):158-165.

2. Carmi L, Tendler A, Bystritsky A, et al. Efficacy and Safety of Deep Transcranial Magnetic Stimulation for Obsessive-Compulsive Disorder: A Prospective Multicenter Randomized Double-Blind Placebo-Controlled Trial. *AJP*. 2019;176(11):931-938. doi:10.1176/appi.ajp.2019.18101180

3. Guzick A.G., Schweissing E., Tendler A., Sheth S.A., Goodman W.K., Storch E.A. Do exposure therapy processes impact the efficacy of deep TMS for obsessive-compulsive disorder? *Journal of Obsessive-Compulsive and Related Disorders*. 2022;35((Guzick, Schweissing, Sheth, Goodman, Storch) Baylor College of Medicine, 1977 Butler Blvd., Houston, TX 77030, United States(Tendler) BrainsWay Inc, 1 Van de Graaff Dr., Burlington, MA 01803, United States):100756. doi:10.1016/j.jocrd.2022.100756

4. Ozer U, Yucens B, Tumkaya S. Efficacy of accelerated deep transcranial magnetic stimulation with double cone coil in obsessive-compulsive disorder: A double-blind, placebo-controlled study. *Journal of Psychiatric Research*. 2024;171:325-331. doi:10.1016/j.jpsychires.2024.02.005

5. Alonso P, Pujol J, Cardoner N, et al. Right prefrontal repetitive transcranial magnetic stimulation in obsessive-compulsive disorder: a double-blind, placebo-controlled study. *Am J Psychiatry*. 2001;158(7):1143-1145. doi:10.1176/appi.ajp.158.7.1143

6. Arumugham SS, Vs S, Hn M, et al. Augmentation Effect of Low-Frequency Repetitive Transcranial Magnetic Stimulation Over Presupplementary Motor Area in Obsessive-Compulsive Disorder: A Randomized Controlled Trial. *J ECT*. 2018;34(4):253-257. doi:10.1097/YCT.0000000000000509

7. Chu L, Wu Y, Yin J, et al. Neurotransmitter system gene variants as biomarkers for the therapeutic efficacy of rTMS and SSRIs in obsessive-compulsive disorder. *Front Psychiatry*. 2024;15:1350978. doi:10.3389/fpsyt.2024.1350978

8. Dutta P, Dhyani M, Garg S, et al. Efficacy of intensive orbitofrontal continuous Theta Burst Stimulation (iOFcTBS) in Obsessive Compulsive Disorder: A Randomized Placebo Controlled Study. *Psychiatry Res*. 2021;298:113784. doi:10.1016/j.psychres.2021.113784

© 2025 Bello D et al. *JAMA Psychiatry*.

9. Elbeh KAM, Elserogy YMB, Khalifa HE, Ahmed MA, Hafez MH, Khedr EM. Repetitive transcranial magnetic stimulation in the treatment of obsessive-compulsive disorders: Double blind randomized clinical trial. *Psychiatry Res*. 2016;238:264-269. doi:10.1016/j.psychres.2016.02.031

10. Fitzgerald PB, Segrave RA, Fornito A, Harrison BJ, Hoy K. A pilot study of fMRI targeted rTMS for obsessive compulsive disorder. *Brain Stimulation*. 2022;15(2):483-484.

11. Fitzsimmons SMDD, Postma T, van Campen AD, et al. TMS-induced plasticity improving cognitive control in OCD I: Clinical and neuroimaging outcomes from a randomised trial of rTMS for OCD. *medRxiv*. Published online November 5, 2023:2023.11.04.23298100. doi:10.1101/2023.11.04.23298100

12. Gomes PV, Brasil-Neto JP, Allam N, Rodrigues de Souza E. A randomized, double-blind trial of repetitive transcranial magnetic stimulation in obsessive-compulsive disorder with three-month follow-up. *J Neuropsychiatry Clin Neurosci*. 2012;24(4):437-443. doi:10.1176/appi.neuropsych.11100242

13. Greenberg BD, George MS, Martin JD, et al. Effect of prefrontal repetitive transcranial magnetic stimulation in obsessive-compulsive disorder: a preliminary study. *Am J Psychiatry*. 1997;154(6):867-869. doi:10.1176/ajp.154.6.867

14. Guo Q, Wang K, Han H, et al. Continuous theta burst stimulation over the bilateral supplementary motor area in obsessive-compulsive disorder treatment: A clinical randomized single-blind sham-controlled trial. *Eur Psychiatry*. 2022;65(1):e64. doi:10.1192/j.eurpsy.2022.2323

15. Harika-Germaneau G, Rachid F, Chatard A, et al. Continuous theta burst stimulation over the supplementary motor area in refractory obsessive-compulsive disorder treatment: A randomized sham-controlled trial. *Brain Stimul*. 2019;12(6):1565-1571. doi:10.1016/j.brs.2019.07.019

16. Hawken ER, Dilkov D, Kaludiev E, Simek S, Zhang F, Milev R. Transcranial Magnetic Stimulation of the Supplementary Motor Area in the Treatment of Obsessive-Compulsive Disorder: A Multi-Site Study. *Int J Mol Sci*. 2016;17(3):420. doi:10.3390/ijms17030420

17. Jahanbakhsh G, Alireza Haji Seyed Javadi S, Majidi M, Khademi M, Karimi R. Effectiveness of adjunctive low-frequency repetitive transcranial magnetic stimulation therapy over the left dorsolateral prefrontal cortex in patients with obsessive-compulsive disorder refractory to medical treatment:A double-blind, randomized clinical. *Asian J Psychiatr*. 2023;80:103384. doi:10.1016/j.ajp.2022.103384

© 2025 Bello D et al. *JAMA Psychiatry*.

18. Jahangard L, Haghighi M, Shyayganfard M, et al. Repetitive Transcranial Magnetic Stimulation Improved Symptoms of Obsessive-Compulsive Disorder, but Also Cognitive Performance: Results from a Randomized Clinical Trial with a Cross-Over Design and Sham Condition. *Neuropsychobiology*. 2016;73(4):224-232. doi:10.1159/000446287

19. Ji GJ, Xie W, Yang T, et al. Pre-supplementary motor network connectivity and clinical outcome of magnetic stimulation in obsessive-compulsive disorder. *Hum Brain Mapp*. 2021;42(12):3833-3844. doi:10.1002/hbm.25468

20. Joshi M., Kar S.K., Dalal P.K. Safety and efficacy of early augmentation with repetitive transcranial magnetic stimulation in the treatment of drug free patients with obsessive-compulsive disorder. *CNS Spectrums*. 2022;((Joshi, Kar, Dalal) Department of Psychiatry, King George's Medical University, Uttar Pradesh, Lucknow, India). doi:10.1017/S1092852922000013

21. Kang JI, Kim CH, Namkoong K, Lee CI, Kim SJ. A randomized controlled study of sequentially applied repetitive transcranial magnetic stimulation in obsessive-compulsive disorder. *J Clin Psychiatry*. 2009;70(12):1645-1651. doi:10.4088/JCP.08m04500

22. Khedr EM, Elbeh K, Saber M, Abdelrady Z, Abdelwarith A. A double blind randomized clinical trial of the effectiveness of low frequency rTMS over right DLPFC or OFC for treatment of obsessive-compulsive disorder. *J Psychiatr Res*. 2022;156:122-131. doi:10.1016/j.jpsychires.2022.10.025

23. Liu W., Shao H., Liao J., Yang D., Ma M., Yang J. Continuous theta-burst stimulation over the right orbitofrontal cortex in treatment-resistant obsessive-compulsive disorder treatment: A randomized sham-controlled trial. *International Journal of General Medicine*. 2021;14((Liu, Liao, Yang) Tianjin Medical University, Tianjin, China(Shao, Yang) Clinical Psychology Department, Tianjin Medical University General Hospital, Tianjin, China(Ma) Tianjin Medical University General Hospital Airport Site, Tianjin, China):3109-3118. doi:10.2147/IJGM.S318069

24. Ma X, Huang Y, Liao L, Jin Y. A randomized double-blinded sham-controlled trial of α electroencephalogram-guided transcranial magnetic stimulation for obsessive-compulsive disorder. *Chin Med J (Engl)*. 2014;127(4):601-606.

25. Mansur CG, Myczkowki ML, de Barros Cabral S, et al. Placebo effect after prefrontal magnetic stimulation in the treatment of resistant obsessive-compulsive disorder: a randomized controlled trial. *Int J Neuropsychopharmacol*. 2011;14(10):1389-1397. doi:10.1017/S1461145711000575

26. Mantovani A, Simpson HB, Fallon BA, Rossi S, Lisanby SH. Randomized sham-controlled trial of repetitive transcranial magnetic stimulation in treatment-resistant obsessive-compulsive disorder. *Int J Neuropsychopharmacol*. 2010;13(2):217-227. doi:10.1017/S1461145709990435

© 2025 Bello D et al. *JAMA Psychiatry*.

27. Mantovani A, Rossi S, Bassi BD, Simpson HB, Fallon BA, Lisanby SH. Modulation of motor cortex excitability in obsessive-compulsive disorder: an exploratory study on the relations of neurophysiology measures with clinical outcome. *Psychiatry Res*. 2013;210(3):1026-1032. doi:10.1016/j.psychres.2013.08.054

28. Meek BP, Fotros A, Abo Aoun M, Modirrousta M. Improvements in error-monitoring and symptoms following low-frequency rTMS of dorsal anterior cingulate cortex in obsessive compulsive disorder; a randomized, sham-controlled study. *Brain Cogn*. 2021;154:105809. doi:10.1016/j.bandc.2021.105809

29. Mukherjee A., Kumre P., Goyal N., Khanra S. Adjunctive neuronavigated accelerated continuous theta-burst stimulation (cTBS) in Obsessive-Compulsive disorder: a randomized sham-controlled study. *CNS Spectrums*. 2022;((Mukherjee, Goyal) Centre for Cognitive Neuroscience, Central Institute of Psychiatry, Ranchi, India(Kumre) Department of Psychiatry, Central Institute of Psychiatry, Ranchi, India(Khanra) Centre for Addiction Psychiatry, Central Institute of Psychiatry,). doi:10.1017/S1092852922000980

30. Naro A, Billeri L, Cannavò A, et al. Theta burst stimulation for the treatment of obsessive-compulsive disorder: a pilot study. *J Neural Transm (Vienna)*. 2019;126(12):1667-1677. doi:10.1007/s00702-019-02098-6

31. Nauczyciel C, Le Jeune F, Naudet F, et al. Repetitive transcranial magnetic stimulation over the orbitofrontal cortex for obsessive-compulsive disorder: a double-blind, crossover study. *Transl Psychiatry*. 2014;4(9):e436. doi:10.1038/tp.2014.62

32. Ni R, Liu Y, Jiang J, et al. Continuous theta burst stimulation to relieve symptoms in patients with moderate obsessive-compulsive disorder: a preliminary study with an external validation. *Transl Psychiatry*. 2024;14(1):1-6. doi:10.1038/s41398-024-03041-4

33. Pelissolo A, Harika-Germaneau G, Rachid F, et al. Repetitive Transcranial Magnetic Stimulation to Supplementary Motor Area in Refractory Obsessive-Compulsive Disorder Treatment: a Sham-Controlled Trial. *Int J Neuropsychopharmacol*. 2016;19(8). doi:10.1093/ijnp/pyw025

34. Prasko J, Pasková B, Záleský R, et al. The effect of repetitive transcranial magnetic stimulation (rTMS) on symptoms in obsessive compulsive disorder. A randomized, double blind, sham controlled study. *Neuro Endocrinol Lett*. 2006;27(3):327-332.

35. Price R.B., Gillan C.M., Hanlon C., et al. Effect of Experimental Manipulation of the Orbitofrontal Cortex on Short-Term Markers of Compulsive Behavior: A Theta Burst Stimulation Study. *The American journal of psychiatry*. 2021;((Price, Gillan, Hanlon, Ferrarelli, Kim, Karim, Renard, Kaskie, Degutis, Wears, Vienneau, Peterchev, Brown, Siegle, Wallace, Ahmari) Department of

© 2025 Bello D et al. *JAMA Psychiatry*.

Psychiatry, University of Pittsburgh, Pittsburgh (Price, Ferrarelli, Kim, Karim, Renard, Kaskie, Degutis, We):appiajp202020060821. doi:10.1176/appi.ajp.2020.20060821

36. Ruffini C, Locatelli M, Lucca A, Benedetti F, Insacco C, Smeraldi E. Augmentation effect of repetitive transcranial magnetic stimulation over the orbitofrontal cortex in drug-resistant obsessive-compulsive disorder patients: a controlled investigation. *Primary Care Companion to the Journal of Clinical Psychiatry*. 2009;11(5):226-230. doi:10.4088/PCC.08m00663

37. Sachdev PS, McBride R, Loo CK, Mitchell PB, Malhi GS, Croker VM. Right versus left prefrontal transcranial magnetic stimulation for obsessive-compulsive disorder: a preliminary investigation. *J Clin Psychiatry*. 2001;62(12):981-984. doi:10.4088/jcp.v62n1211

38. Sachdev PS, Loo CK, Mitchell PB, McFarquhar TF, Malhi GS. Repetitive transcranial magnetic stimulation for the treatment of obsessive compulsive disorder: a double-blind controlled investigation. *Psychol Med*. 2007;37(11):1645-1649. doi:10.1017/S0033291707001092

39. Sarkhel S, Sinha VK, Praharaj SK. Adjunctive high-frequency right prefrontal repetitive transcranial magnetic stimulation (rTMS) was not effective in obsessive–compulsive disorder but improved secondary depression. *Journal of Anxiety Disorders*. 2010;24(5):535-539.

40. Seo HJ, Jung YE, Lim HK, Um YH, Lee CU, Chae JH. Adjunctive low-frequency repetitive transcranial magnetic stimulation over the right dorsolateral prefrontal cortex in patients with treatment-resistant obsessive-compulsive disorder: A randomized controlled trial. *Clinical Psychopharmacology and Neuroscience*. 2016;14(2):153-160.

41. Vidya KL, Rao PG, Goyal N. Adjuvant Priming Repetitive Transcranial Magnetic Stimulation for Treatment-Resistant Obsessive-Compulsive Disorder: In Search of a New Paradigm! *J ECT*. 2022;38(1):e1-e8. doi:10.1097/YCT.0000000000000791

42. Zhang K, Fan X, Yuan J, et al. Impact of serotonin transporter gene on rTMS augmentation of SSRIs for obsessive compulsive disorder. *Neuropsychiatric Disease and Treatment*. 2019;15. http://proxy.library.vanderbilt.edu/login?url=https://www.proquest.com/docview/2541899624?accountid=14816&bdid=30121&_bd=rm%2FESvL%2FkuSoRvo65oxH5CpgWJ4%3D

43. Ziblak A., Tumkaya S., Kashyap H. Transcranial magnetic stimulation over orbitofrontal cortex in obsessive compulsive disorder: A double-blind placebo-controlled trial. *Journal of Obsessive-Compulsive and Related Disorders*. 2021;31((Ziblak) Psychiatry Clinic, Denizli State Hospital, Denizli, Turkey(Tumkaya) Department of Psychiatry, Pamukkale University, Faculty of Medicine,

© 2025 Bello D et al. *JAMA Psychiatry*.

Denizli, Turkey(Kashyap) Department of Clinical Psychology, National Institute of Mental Health and Neuro Sci):100687. doi:10.1016/j.jocrd.2021.100687

44. Zou J, Wu S, Yuan X, Hu Z, Tang J, Hu M. Effects of acceptance and commitment therapy and repetitive transcranial magnetic stimulation on obsessive–compulsive disorder. *Frontiers in Psychiatry*. 2022;12. http://proxy.library.vanderbilt.edu/login?url=https://www.proquest.com/docview/2635263685?accountid=14816&bdid=30121&_bd=Aah24VjqiDjOfSiQZrx2VIuj7vQ%3D

45. Addicott MA, Kinney KR, Saldana S, et al. A randomized controlled trial of intermittent theta burst stimulation to the medial prefrontal cortex for tobacco use disorder: Clinical efficacy and safety. *Drug Alcohol Depend*. 2024;258:111278. doi:10.1016/j.drugalcdep.2024.111278

46. Amiaz R, Levy D, Vainiger D, Grunhaus L, Zangen A. Repeated high-frequency transcranial magnetic stimulation over the dorsolateral prefrontal cortex reduces cigarette craving and consumption. *Addiction*. 2009;104(4):653-660. doi:10.1111/j.1360-0443.2008.02448.x

47. Bellini BB, Scholz JR, Abe TO, et al. Does deep TMS really works for smoking cessation? A prospective, double blind, randomized, sham controlled study. *Prog Neuropsychopharmacol Biol Psychiatry*. 2024;132:110997. doi:10.1016/j.pnpbp.2024.110997

48. Dinur-Klein L, Dannon P, Hadar A, et al. Smoking cessation induced by deep repetitive transcranial magnetic stimulation of the prefrontal and insular cortices: a prospective, randomized controlled trial. *Biol Psychiatry*. 2014;76(9):742-749. doi:10.1016/j.biopsych.2014.05.020

49. Li X, Hartwell KJ, Owens M, et al. Repetitive transcranial magnetic stimulation of the dorsolateral prefrontal cortex reduces nicotine cue craving. *Biol Psychiatry*. 2013;73(8):714-720. doi:10.1016/j.biopsych.2013.01.003

50. Li X, Du L, Sahlem GL, Badran BW, Henderson S, George MS. Repetitive transcranial magnetic stimulation (rTMS) of the dorsolateral prefrontal cortex reduces resting-state insula activity and modulates functional connectivity of the orbitofrontal cortex in cigarette smokers. *Drug Alcohol Depend*. 2017;174:98-105. doi:10.1016/j.drugalcdep.2017.02.002

51. Li X, Hartwell KJ, Henderson S, Badran BW, Brady KT, George MS. Two weeks of image-guided left dorsolateral prefrontal cortex repetitive transcranial magnetic stimulation improves smoking cessation: A double-blind, sham-controlled, randomized clinical trial. *Brain Stimul*. 2020;13(5):1271-1279. doi:10.1016/j.brs.2020.06.007

© 2025 Bello D et al. *JAMA Psychiatry*.

52. Li X, Toll BA, Carpenter MJ, Nietert PJ, Dancy M, George MS. Repetitive Transcranial Magnetic Stimulation for Tobacco Treatment in Cancer Patients: A Preliminary Report of a One-Week Treatment. *Journal of Smoking Cessation*. Published online 2022:1-6. doi:10.1155/2022/2617146

53. Li S, Ma X, Chen H, et al. rTMS effects on urges and severity of tobacco use disorder operate independently of a retrieval-extinction component and involve frontal-striatal pathways. *J Affect Disord*. 2024;349:21-31. doi:10.1016/j.jad.2024.01.048

54. Marques RC, Marques D, Vieira L, Cantilino A. Left frontal pole repetitive transcranial magnetic stimulation reduces cigarette cue-reactivity in correlation with verbal memory performance. *Drug Alcohol Depend*. 2022;235:109450. doi:10.1016/j.drugalcdep.2022.109450

55. Mikellides G., Michael P., Psalta L., Stefani A., Schuhmann T., Sack A.T. Accelerated Intermittent Theta Burst Stimulation in Smoking Cessation: Placebo Effects Equal to Active Stimulation When Using Advanced Placebo Coil Technology. *Frontiers in Psychiatry*. 2022;13((Mikellides, Schuhmann, Sack) Department of Cognitive Neuroscience, Faculty of Psychology and Neuroscience, Maastricht University, Maastricht, Netherlands(Mikellides, Michael) Cyprus rTMS Centre, Larnaca, Cyprus(Psalta) Department of Psychology, Universit):892075. doi:10.3389/fpsyt.2022.892075

56. Pripfl J, Tomova L, Riecansky I, Lamm C. Transcranial magnetic stimulation of the left dorsolateral prefrontal cortex decreases cue-induced nicotine craving and EEG delta power. *Brain Stimulation*. 2014;7(2):226-233.

57. Rose JE, McClernon FJ, Froeliger B, Behm FM, Preud'homme X, Krystal AD. Repetitive transcranial magnetic stimulation of the superior frontal gyrus modulates craving for cigarettes. *Biol Psychiatry*. 2011;70(8):794-799. doi:10.1016/j.biopsych.2011.05.031

58. Zangen A, Moshe H, Martinez D, et al. Repetitive transcranial magnetic stimulation for smoking cessation: a pivotal multicenter double-blind randomized controlled trial. *World Psychiatry*. 2021;20(3):397-404. doi:10.1002/wps.20905

59. Abdelrahman AA, Noaman M, Fawzy M, Moheb A, Karim AA, Khedr EM. A double-blind randomized clinical trial of high frequency rTMS over the DLPFC on nicotine dependence, anxiety and depression. *Sci Rep*. 2021;11(1):1640. doi:10.1038/s41598-020-80927-5

60. Dieler AC, Dresler T, Joachim K, Deckert J, Herrmann MJ, Fallgatter AJ. Can intermittent theta burst stimulation as add-on to psychotherapy improve nicotine abstinence? Results from a pilot study. *Eur Addict Res*. 2014;20(5):248-253. doi:10.1159/000357941

© 2025 Bello D et al. *JAMA Psychiatry*.

61. Du X, Choa FS, Chiappelli J, et al. Combining neuroimaging and brain stimulation to test alternative causal pathways for nicotine addiction in schizophrenia. *Brain Stimul*. 2024;17(2):324-332. doi:10.1016/j.brs.2024.02.020

62. Huang W, Shen F, Zhang J, Xing B. Effect of Repetitive Transcranial Magnetic Stimulation on Cigarette Smoking in Patients with Schizophrenia. *Shanghai Arch Psychiatry*. 2016;28(6):309-317. doi:10.11919/j.issn.1002-0829.216044

63. Ibrahim C, Tang VM, Blumberger DM, et al. Efficacy of insula deep repetitive transcranial magnetic stimulation combined with varenicline for smoking cessation: A randomized, double-blind, sham controlled trial. *Brain Stimulation*. 2023;16(5):1501-1509. doi:10.1016/j.brs.2023.10.002

64. Lechner WV, Philip NS, Kahler CW, Houben K, Tirrell E, Carpenter LL. Combined Working Memory Training and Transcranial Magnetic Stimulation Demonstrates Low Feasibility and Potentially Worse Outcomes on Delay to Smoking and Cognitive Tasks: A Randomized 2 × 2 Factorial Design Pilot and Feasibility Study. *Nicotine Tob Res*. 2022;24(12):1871-1880. doi:10.1093/ntr/ntac183

65. Li X, Caulfield KA, Hartwell KJ, Henderson S, Brady KT, George MS. Reduced executive and reward connectivity is associated with smoking cessation response to repetitive transcranial magnetic stimulation: A double-blind, randomized, sham-controlled trial. *Brain Imaging Behav*. 2024;18(1):207-219. doi:10.1007/s11682-023-00820-3

66. Moeller SJ, Gil R, Weinstein JJ, et al. Deep rTMS of the insula and prefrontal cortex in smokers with schizophrenia: Proof-of-concept study. *Schizophr*. 2022;8(1):1-9. doi:10.1038/s41537-022-00224-0

67. Newman-Norlund RD, Gibson M, McConnell PA, Froeliger B. Dissociable effects of theta-burst repeated transcranial magnetic stimulation to the inferior frontal gyrus on inhibitory control in nicotine addiction. *Frontiers in Psychiatry*. 2020;11. http://proxy.library.vanderbilt.edu/login?url=https://www.proquest.com/docview/2719024920?accountid=14816&bdid=30121&_bd=S652gy%2FEXDCxNAgFO%2BW2N7ReF7o%3D

68. Sheffer CE, Mennemeier M, Landes RD, et al. Neuromodulation of delay discounting, the reflection effect, and cigarette consumption. *Journal of Substance Abuse Treatment*. 2013;45(2):206-214. doi:10.1016/j.jsat.2013.01.012

69. Sheffer C.E., Bickel W.K., Brandon T.H., et al. Preventing relapse to smoking with transcranial magnetic stimulation: Feasibility and potential efficacy. *Drug and Alcohol Dependence*. 2018;182((Sheffer, Deen, Panissidi, Abdali, Pittman, Lunden, Prashad, Malhotra, Mantovani) City University of New York School of Medicine, City College of New York, Harris Hall, 160 Convent Ave, New York, NY 10031, United States(Sheffer) Roswell Park Cancer Instit):8-18. doi:10.1016/j.drugalcdep.2017.09.037

© 2025 Bello D et al. *JAMA Psychiatry*.

70. Trojak B, Meille V, Achab S, et al. Transcranial Magnetic Stimulation Combined With Nicotine Replacement Therapy for Smoking Cessation: A Randomized Controlled Trial. *Brain Stimul*. 2015;8(6):1168-1174. doi:10.1016/j.brs.2015.06.004

71. Wing VC, Bacher I, Wu BS, Daskalakis ZJ, George TP. High frequency repetitive transcranial magnetic stimulation reduces tobacco craving in schizophrenia. *Schizophr Res*. 2012;139(1-3):264-266. doi:10.1016/j.schres.2012.03.006

© 2025 Bello D et al. *JAMA Psychiatry*.
