# Supplementary material for: Symptom Provocation and Clinical Response to Transcranial Magnetic Stimulation: A Systematic Review and Meta-Analysis
Source: JAMA Psychiatry. 2025 Jun 4;82(8):768–77. doi: 10.1001/jamapsychiatry.2025.0792 (PMC12138803; doi:10.1001/jamapsychiatry.2025.0792)
Supplement: Supplement 2. — Data Sharing Statement. [file jamapsychiatry-e250792-s002.pdf]

Data Sharing Statement

Bello. Symptom Provocation and Clinical Response to Transcranial Magnetic Stimulation.
JAMA Psychiatry. Published June 04, 2025. doi:10.1001/jamapsychiatry.2025.0792

Data
Data available: No
